# Human dorsal forebrain organoids show differentiation-state-specific protein secretion

**DOI:** 10.1016/j.isci.2025.112935

**Published:** 2025-06-19

**Authors:** Zeynep Yentür, Theresa Kagermeier, Kseniia Sarieva, Mohamed A. Jarboui, Katharina Becker, Simone Mayer

**Affiliations:** 1 The Heidelberg Academy of Sciences and Humanities, Heidelberg, Germany; 2 Hertie Institute for Clinical Brain Research, University of Tübingen, Tübingen, Germany; 3 International Max Planck Research School, Graduate Training Centre of Neuroscience, University of Tübingen, Tübingen, Germany; 4 Graduate Training Centre of Neuroscience, University of Tübingen, Tübingen, Germany; 5 Core Facility for Medical Proteomics, Institute for Ophthalmic Research, University Clinic Tübingen, Tübingen, Germany; 6 Zoological Institute, Karlsruhe Institute of Technology (KIT), Karlsruhe, Germany; 7 Institute of Biological and Chemical Systems - Functional Molecular Systems, Karlsruhe Institute of Technology (KIT), Karlsruhe, Germany

**Keywords:** Natural sciences, Biological sciences, Neuroscience, Tissue engineering

## Abstract

The human brain microenvironment undergoes dynamic changes during development, which have been incompletely characterized in *in vitro* models including neural organoids. Here, we used liquid chromatography-mass spectrometry to investigate proteome and secretome changes in human dorsal forebrain organoids derived from three hiPSC lines at days 20, 35, and 50 of differentiation. Proteome and immunohistochemical analysis revealed reduced proliferation and increased differentiation of progenitor cells gradually over time. In contrast, secretome analysis showed distinct characteristics at each timepoint — notably, at day 35, the numbers of cell adhesion molecules, synaptic proteins, and proteases were increased. Taken together, we present a resource describing the dynamic features of a neural organoid proteome and secretome across different genetic backgrounds. We describe the unique niche composition of neural organoids during the period of neurogenesis and suggest that synaptic proteins may play a role in guiding neurogenesis.

## Highlights

• Proteomic analysis of DFOs on three time points shows neural differentiation
• Protein secretion increases during peak neurogenesis at D35 and D50
• Cell adhesion molecules, synapse proteins, and metalloproteases are mainly secreted at D35
• Extracellular matrix proteins are predominantly secreted at D50

## Graphical abstract

**Figure:**
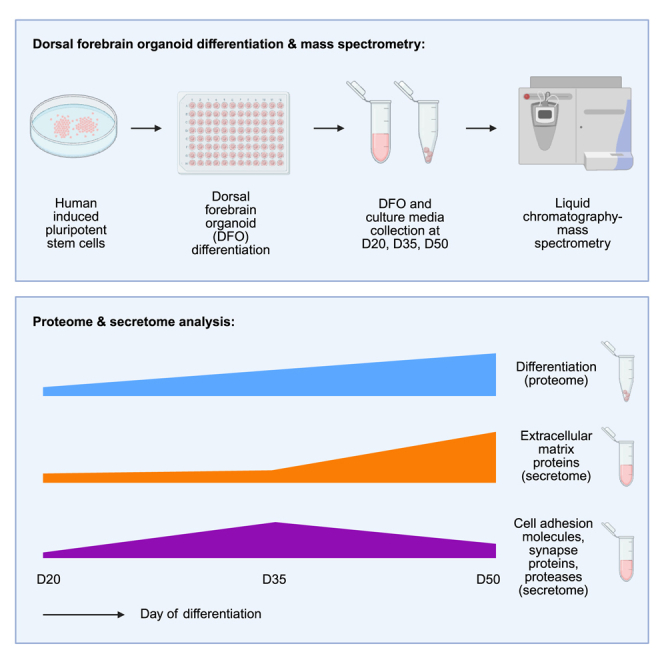

## Introduction

Human neocortical development follows a precise trajectory determined by complex interactions between genes and the cellular environment.^1^^,^^2^ The specific environments that cells reside in are called niches. These specialized microenvironments have a dynamic and complex combination of extracellular components, including extracellular matrix (ECM) and cell adhesion molecules.^3^^,^^4^ Disruptions in these extracellular components can lead to neurodevelopmental and neurological disorders including autism spectrum disorder (ASD), schizophrenia, bipolar disorder, and lissencephaly,^4^^,^^5^ further highlighting their importance. In the human neocortex, ECM expression dynamically changes in the cortical wall throughout development.^5^ For example, in germinal zones ECM components promote cell proliferation, while in the cortical plate they promote neuronal maturation.^6^ This indicates the dynamic role of the ECM in different layers of the neocortex. Therefore, longitudinal studies are needed to characterize the dynamic cellular microenvironment in the prenatal human brain. As human brain development is difficult to study *in vivo*, different models are used to study it *in vitro*.

*In vitro* tools, such as neural organoids, provide a three-dimensional (3D) model to study early human brain development by recapitulating the molecular, cellular, and cytoarchitectural complexity of the brain.^7^^,^^8^^,^^9^^,^^10^^,^^11^ Neural organoids, derived from human brain tissue or human pluripotent stem cells (hPSCs), have also been used to model human-specific microenvironments.^12^^,^^13^^,^^14^ For example, human fetal brain tissue-derived organoids showed *in vitro* establishment of an ECM niche that recapitulates its *in vivo* counterpart over several months.^12^ In another study, hPSC-derived neural organoids were cultured with decellularized human brain tissue-derived ECM, increasing the number of radial glia cells and enhancing neurogenesis compared to Matrigel supplementation.^13^ While these *in vitro* studies give valuable insights into the microenvironment of the developing human brain, the availability of *ex vivo* human brain tissue is limited. Due to the greater accessibility and potential scalability of hPSC cultures, most researchers rely solely on hPSC-derived neural organoids. Recently, Martins-Costa et al. demonstrated that hPSC-derived neural organoids endogenously produced and self-assembled ECM in the early developmental stages and differentiated to forebrain lineage in the absence of exogenous Matrigel supplementation.^14^ Therefore, hPSC-derived neural organoids are a promising approach to model the developing human brain microenvironment.

While transcriptomic analysis is often the preferred readout, neural organoid studies have demonstrated the importance of proteomics due to the nonlinear relationship between protein levels and RNA expression that can be attributed to posttranscriptional gene regulatory mechanisms.^15^^,^^16^^,^^17^ Mass spectrometry (MS) has been performed to characterize both the proteome and the secretome of neural organoids^18^^,^^19^ allowing to understand their relationship. For example, proteome and secretome analyses were used in combination to identify the role of ECE2 (a protease localized in secretory vesicles) in the secretion of ECM proteins and their effect on neural migration in the neocortex.^18^ Additionally, *de novo* variants in the ECM gene LGALS3BP were shown to disrupt the secretome of neural organoids.^19^ A follow-up study demonstrated that LGALS3BP regulates cellular differentiation via extracellular vesicles and that mutations in LGALS3BP altered the protein composition of these vesicles.^20^ This extracellular vesicle study and previous whole secretome studies were limited to a single time point and were mainly focused on mutant vs. control comparison.^18^^,^^19^^,^^20^ More recently, Forero et al. investigated the temporal dynamics of the content of extracellular vesicles and their role in neurodevelopment of trafficking key molecules in a cell type–specific manner.^16^ These studies show that the secretome has a biological function in neurodevelopment, and its alterations have functional implications in neurodevelopmental disorders.

Extending previous studies, we performed whole organoid proteome and secretome analyses of regionalized neural organoids called dorsal forebrain organoids (DFOs)^21^^,^^22^ over a developmental period using three cell lines and captured the dynamic nature of the brain microenvironment. This approach allowed the identification of protein-level characteristics unique to different developmental stages in a genetic background-independent manner. We found distinct protein dynamics in the organoid proteome and secretome: While the proteome showed signatures of increasing neural differentiation gradually over time, secretome showed distinct characteristics at each timepoint, with a specific increase in adhesion molecules, synaptic proteins, and proteases during peak neurogenesis. Our study lays the foundation for studying the functional roles of synaptic protein secretion during neurogenesis.

## Results

**Figure 1:**
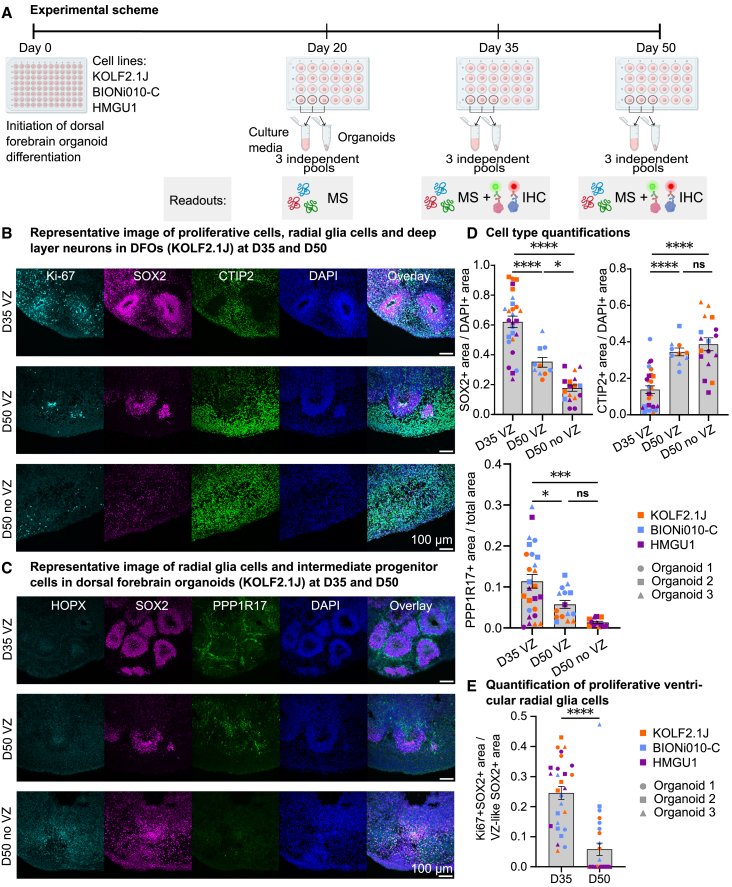
The cell type composition changes from proliferative radial glia cells to differentiated neurons over time as dorsal forebrain organoids mature (A) The experimental scheme for dorsal forebrain organoid plating and collection of samples for mass spectrometry (MS) and immunohistochemistry (IHC). On D20, D35, and D50, culture media and organoids from three individual samples were pooled and snap-frozen in liquid nitrogen for MS analysis. On D35 and D50, three organoids were fixed for IHC. (B) Dorsal forebrain organoids contain VZ-like regions visualized by Ki67 positive proliferative cells (cyan) and dense SOX2 positive structures (magenta) surrounded by CTIP2 positive deep layer neurons (green). The same markers were used for organoids that did not contain VZ-like structures at D50. Representative images are shown to visualize different cell types, representative organoids are generated from the KOLF2.1J cell line. The scale bar represents 100 μm. (C) Dorsal forebrain organoids contain intermediate progenitor cells visualized by PPP1R17 positive cells (green) at D35 and D50. PPP1R17 positive cells surround the outer layer of the VZ-like region visualized by SOX2 positive cells (magenta). Outer radial glial cells are visualized by HOPX-positive cells (cyan). The same markers were used for organoids that did not contain VZ-like structures at D50. Representative images are shown to visualize different cell types, representative organoids are generated from the KOLF2.1J cell line. The scale bar represents 100 μm. (D) Quantification of SOX2-positive (top, left) and CTIP2-positive (top, right) area over DAPI-positive area. D35 VZ (*n* = 27 for SOX2 and CTIP2), D50 VZ (*n* = 10 SOX2 and CTIP2), and D50 no VZ (*n* = 17 for SOX2 and CTIP2). Quantification of PPP1R17-positive area over total area (bottom). D35 VZ (*n* = 26), D50 VZ (*n* = 15) and D50 no VZ (*n* = 12). Data points represent individual quantifications. (E) Quantification of Ki67 and SOX2 double-positive proliferative ventricular radial glia cells (vRGs) (bottom). Organoids that contained VZ-like regions were quantified and organoids that do not have VZ-like regions were plotted and analyzed as 0, D35 VZ (*n* = 27) and D50 (*n* = 27). For quantification (D) and (E), bars represent the mean of each quantification, and error bars represent ±SEM. Aligned Rank Transform (ART) ANOVA was used to compare the conditions as most of the data failed the normality test. The color of the data points represents cell line, KOLF2-1J, orange; BIONi010-C, green; HMGU1, purple, one batch per cell line was quantified. The shape of the data points represents individual organoids, three organoids per cell line were quantified. For quantification (D) Tukey's test was performed for multiple comparisons between day conditions. ns, non-significant *p*-value >0.05; ∗ *p*-value <0.05; ∗∗ *p*-value <0.01; ∗∗∗ *p*-value <0.001; ∗∗∗∗ *p*-value <0.0001. Illustrations for experimental schemes in (A) created with BioRender.com. See also Figure S1.

To reveal differentiation trajectories of DFOs at the proteome and secretome levels, human DFOs were differentiated according to a previously published protocol^8^ using three control human iPSC lines (KOLF2.1J, BIONi010-C, and HMGU1). Organoids were collected for different analyses such as immunohistochemistry (IHC), proteome, and secretome analyses at day (D)20, D35 and D50 (Figure 1A).

### DFOs are composed of main progenitor cell types that mature into excitatory neurons over time

Cell-type composition in the neocortex is dynamic throughout development.^23^ Since MS-based analyses are bulk readouts, to interpret the data, it is important to understand the underlying cellular composition changes. To visualize the main cell types and to understand the cellular composition dynamics prior to proteomic analysis, we performed IHC at D35 and D50 on three organoids per cell line per time point (Figures 1B and 1C). Ventricular zone (VZ)-like regions, which are also referred to as neural rosettes, are the main proliferative zones in DFOs and emerge early in organoid development.^24^ At D35 of differentiation, all DFOs had at least three VZ-like regions while at D50 some of the organoids did not have VZ-like regions (Figures 1B and 1C). This is likely due to the increased radial glia (RG) differentiation at D50.^25^ We will refer to these organoids as DFOs without VZ-like regions in the remainder of this study. We quantified changes in different cell populations over time (Figure S1). SOX2-positive RG and PPP1R17-positive intermediate progenitor cell (IPC) populations were significantly decreased in D50 compared to D35, while the CTIP2-positive deep layer neuronal population was increased in D50 compared to D35 (Figure 1D). A significant difference was observed between D50 DFOs with VZ-like regions and D50 DFOs without VZ-like regions for SOX2-positive cells but not for other cell types (Figure 1D). DFOs without VZ-like regions had a significantly smaller proportion of SOX2-positive area than DFOs with VZ-like regions (Figure 1D). Next, we focused on the VZ-like regions by isolating the rosette structures in the acquired images and quantified the proliferative ventricular radial glia (vRG) cells (Figure S1C). Proliferating ventricular RGs (Ki67+, SOX2+) were reduced at D50 compared to D35 (Figure 1E). Finally, to check whether there are cell line specific changes in the cellular composition, we split the data points based on different cell lines, KOLF2.1J, BIONi010-C and HMGU1 (Figures S2A–S2D). Similar trends were observed in all three cell lines: the SOX2+ progenitor population was reduced and deep layer CTIP2+ neuronal population was increased over time (Figures S2A–S2D). In line with previous neural organoid studies,^23^ our IHC findings demonstrated that DFOs matured from D35 to D50 by increasing deep layer neuronal populations and decreasing proliferative vRGs and IPCs.

### Proteomic analysis revealed reduced proliferative capacity and an increased abundance of synapse-related markers

**Figure 2:**
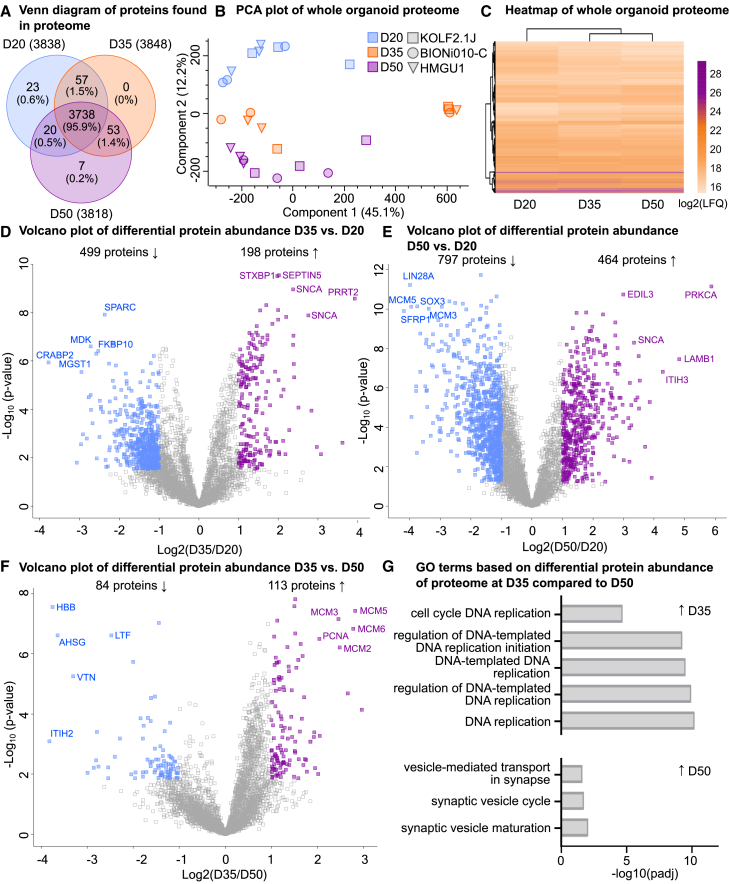
Whole organoids proteome analysis of dorsal forebrain organoids at D20, D35, and D50 reveals reduced proliferation and increased synapse-related proteins over time (A) Venn diagram of common and unique proteins found in each time point, to be included proteins must be present in at least one sample, before data imputation. Repeating proteins were counted once. (B) Principal component analysis of individual whole organoid samples from three cell lines at D20 (blue), D35 (orange), and D50 (purple). Before data imputation. (C) Hierarchical clustering of all abundant proteins using averaged log2 transformed LFQ values for each time point. Average of nine samples from three cell lines (KOLF2.1J, BIONi010-C, and HMGU1, *n* = 3 for each cell line) per time point. (D) Volcano plots visualizing differential protein abundance at D35 vs. D20. (E) Volcano plots visualizing differential protein abundance at D50 vs. D20. (F) Volcano plots visualizing differential protein abundance at D35 vs. D50. (G) GO biological processes analysis of proteins with increased (top) and decreased abundance (bottom) from D35 vs. D50 proteome analysis. For (D), (E), and (F), the difference between averaged log2 transformed LFQ values plotted as log2 fold change against -log(*p*-value) for each protein detected and proteins that pass the significance cutoff (FDR<0.05 log_2_fold change values ≤ -1 and ≥1) are visualized in color. Proteins with decreased abundance (blue), proteins with increased abundance (purple), and nonsignificant proteins (gray) are visualized. See also Figures S2A and S2B.

To investigate the cellular states and the intrinsic protein expression of DFOs over time we performed liquid chromatography-mass spectrometry (LC-MS). Proteomic analysis was performed on 27 samples. Each sample consisted of 3 pooled organoids, in triplicates for each cell line (KOLF2.1J, BIONi010-C, and HMGU1) collected on D20, D35, and D50 (Figure 1A). A total of 4431 proteins were identified (Table S2), 95.9% of them were identified across all time points. This indicates that protein diversity across time points was similar. D20 and D50 organoids had distinctly identified proteins (23 and 7 proteins, respectively) (Figure 2A; Table S2). At D35 there were no uniquely expressed proteins, but 57 proteins were shared with D20 only and 53 proteins with D50 only. This indicates the protein signature at D35 is transitionary between D20 and D50. We performed dimensionality reduction using principal components analysis (PCA) on individual samples and confirmed distinct clustering of samples based on their day of differentiation in component 2 (Figure 2B). Importantly, cluster separation was not driven by cell lines in component 1 or component 2 (Figure 2B). To check whether there are any differences in the proteome across different genetic backgrounds, we performed differential protein abundance analysis comparing the three cell lines at D20, D35, and D50 after imputation of the missing values (Figure S3A). Similar to the PCA plot, we did not identify differences in the proteomes of DFOs generated from KOLF2.1J, BIONi010-C, and HMGU1 cell lines (Figure S3A). Next, the similarity between organoid proteomes at different time points was visualized using hierarchical clustering of all identified proteins (Figure 2C; Table S3). While proteomes were similar overall, the highest similarity was found between D35 and D50 proteomes.

To identify changes in protein abundance over time, we performed differential protein abundance analysis, using significance threshold false discovery rate (FDR)-corrected q < 0.05 and log_2_fold change values ≤ -1 and ≥1 (Figures 2D–2F). The analysis of D35 vs. D20 proteomes yielded 499 proteins with decreased abundance at D35 and 198 proteins with increased abundance at D35 (Figure 2D). D50 vs. D20 yielded 797 proteins with decreased abundance at D50 and 464 proteins with increased abundance at D50 (Figure 2E), and D35 vs. D50 yielded 84 proteins with decreased abundance at D35 and 113 proteins with increased abundance at D35 (Figure 2F). Functional annotations of these proteins were carried out using gene ontology (GO) biological processes (BP) analysis. D35 vs. D20 comparison revealed enrichment of terms related to “DNA replication” in D20 organoids and enrichment of terms related to synapses in D35 organoids (Figure S4A). Likewise, in a D50 vs. D20 comparison, cell division, and DNA replication-related terms were enriched for D20, and synapse-related terms were enriched in D50 organoids (Figure S4B). The comparison of D35 to D50 showed enrichment of DNA replication at D35 and enrichment of synapse-related terms at D50 (Figure 2G). Therefore, the organoid IHC analysis indicates that as neurons are formed, progenitor cell proliferation decreases (Figure 1), congurently the organoid proteome shows increasingly neuronal and decreasingly proliferative signatures (Figure 2).

### Secretome analysis revealed developmental stage-dependent changes in secreted proteins

**Figure 3:**
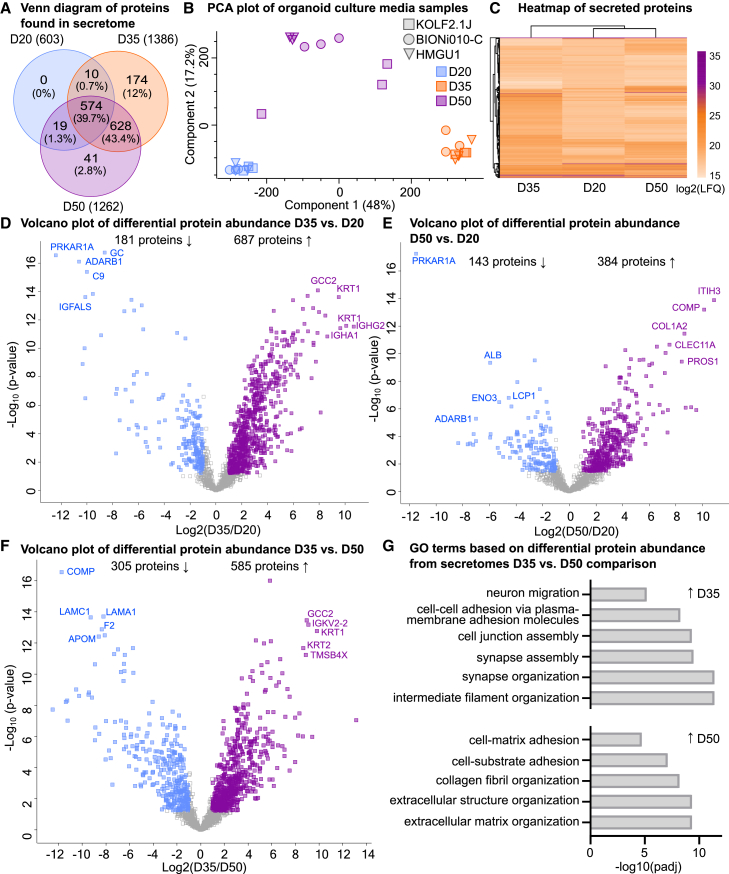
Organoid secretome analysis of dorsal forebrain organoids at D20, D35, and D50 reveals a non-chronological order of proteins secreted to extracellular space (A) Venn diagram of proteins found in the organoid secretome at each time point and proteins found in at least one organoid culture media were included, before data imputation. Repeating proteins were counted once. (B) Principal components analysis of all samples from D20 (blue), D35 (orange), and D50 (purple). Each point represents one sample. Before data imputation. (C) Hierarchical clustering of all abundant proteins at different time points using averaged log2 transformed LFQ values of samples. Three cell lines (KOLF2.1J, BIONi010-C, and HMGU1) with three pooled samples each, *n* = 9 per time point. (D) Volcano plot of significant proteins with increased abundance (purple) and decreased abundance (blue) proteins comparing D35 vs. D20 cell culture media samples. (E) Volcano plot of significant proteins with increased abundance (purple) and decreased abundance (blue) proteins comparing D50 vs. D20 cell culture media samples. (F) Volcano plot of significant proteins with increased abundance (purple) and decreased abundance (blue) proteins comparing D35 vs. D50 cell culture media samples. (G) GO biological processes analysis of proteins with increased abundance (top) and decreased abundance (bottom) found in D35 vs. D50 secretome analysis. For (D), (E), and (F), the difference between time points is plotted against -log(P-value) with significance cutoff (FDR<0.05, log_2_fold change values ≤ -1 and ≥1). See also Figures S2C and S2D.

To investigate the changes in secreted proteins and changes in niche composition, we collected organoid culture media on D20, D35, and D50 from the same organoids harvested for proteome analysis (Figure 2). In organoid culture media, 1610 proteins were identified (Table S2). Compared to the proteome, the secretome had a lower percentage of proteins identified at all time points (95.9% for proteome, 39.7% for secretome) (Figure 3A). In total, at D20 there were 603 secreted proteins, at D35 1386 secreted proteins, and at D50 1262 secreted proteins. Therefore, protein secretion increases as neurogenesis proceeds at D35 and D50 compared to D20 (Figure 3A). Comparison of the secretome protein profiles at the different time points revealed that D20 organoids did not secrete unique proteins compared to later developmental time points (Figure 3A). On the contrary, D35 and D50 organoids secreted distinct proteins, with D35 having the highest number of these (0 distinct proteins at D20, 174 distinct proteins at D35, and 41 distinct proteins at D50). The PCA plot of individual culture media samples displayed distinct clustering dependent on time points (Figure 3B). Differential protein abundance analysis comparing three cell lines at D20, D35, and D50 did not indicate a significant difference between secretomes of DFOs generated from KOLF2.1J, BIONi010-C, and HMGU1 cell lines (Figure S3B). Hierarchical clustering after data imputation revealed secretome signatures at D20 and D50 were more alike than the secretome signatures at D35 (Figure 3C; Table S3). This indicates unique features of different developmental stages instead of the linear progression that was seen in the proteome.

Next, we analyzed the changes in secretome protein abundance over time by pairwise comparisons between the time points (FDR-corrected q < 0.05 and log_2_fold change values ≤ -1 and ≥1) (Figures 3D–3F). The comparison of D35 to D20 secretome yielded 687 proteins with increased abundance at D35 and 181 proteins with decreased abundance at D35 (Figure 3D) and 384 proteins with increased abundance at D50 and 143 proteins with decreased abundance at D50 in the D50 to D20 comparison (Figure 3E). Finally, the secretome comparison of D35 to D50 yielded 585 proteins with increased abundance at D35 and 305 proteins with decreased abundance at D35 (Figure 3F). To understand the functional implications, we performed GO BP. We found enrichment of terms related to cell adhesion at D35 in comparison with D20 (Figure S4C), while the D50 vs. D20 comparison highlighted enrichment of terms related to ECM at D50 (Figure S4D). Similar to the organoid proteome, synapse-related terms were enriched for both D35 vs. D20 and D50 vs. D20 comparisons (Figure S4C and S4D). Additionally, the D20 secretome was enriched with proteins involved in the negative regulation of peptidase activity in the D35 vs. D20 comparison (Figure S4C) and the D20 secretome was enriched with intermediate filaments proteins in the D50 vs. D20 comparison (Figure S4D). We further found an increased abundance of cell adhesion-related proteins, including synapse assembly related proteins, as well as ECM proteases at D35 compared to D50 (Figure 3G). In the D50 secretome, ECM and matrix adhesion-related terms were more prominent than in the D35 secretome (Figure 3G). These findings highlight the unique features detected in the secretome profile of DFOs as progenitor cells differentiate, especially at D35 and D50, where the protein profile was distinct with the presence of cell adhesion molecules at D35 and ECM proteins at D50.

### D35 secretome shows a distinct signature with increased abundance of synaptic adhesion molecules and cell adhesion molecules compared to the D50 secretome

**Figure 4:**
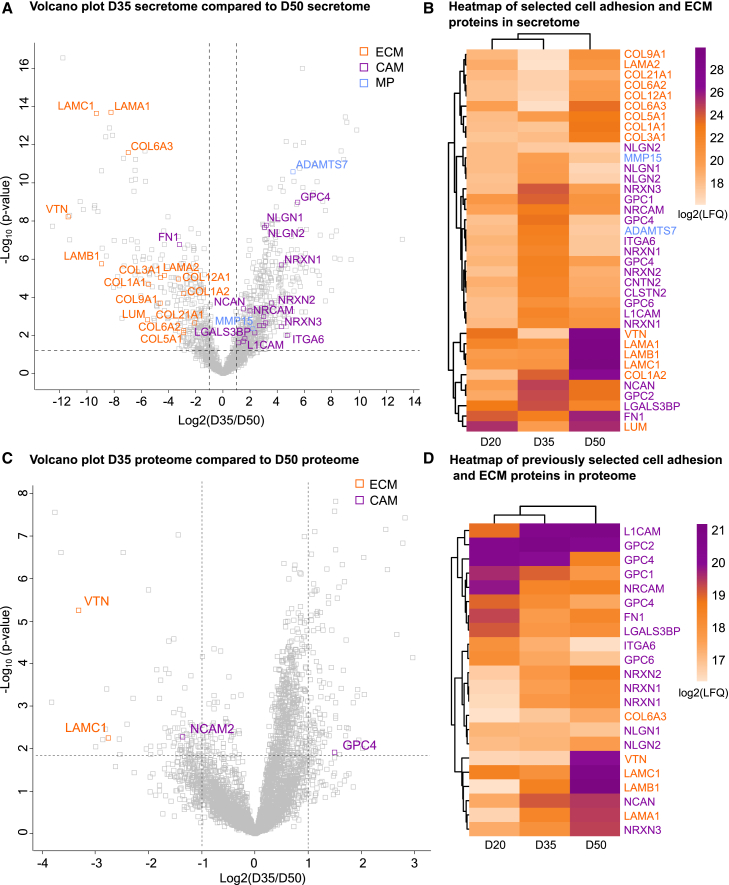
Differential secretome analysis reveals a unique protein signature of D35 dorsal forebrain organoids compared to D50 which is not captured in whole organoid proteome analysis (A) Volcano plot comparing D35 vs. D50 secretome. Significantly deregulated proteins visualized above the cutoff line (FDR<0.05, log_2_fold change values ≤ -1 and ≥1) with selected proteins showing names including extracellular matrix proteins (ECM, orange), cell adhesion molecules (CAM, purple) and metalloproteinases (MP, blue). (B) Heatmap of selected proteins from A using averaged log2 transformed LFQ values of secretome analysis at D20, D35, and D50 (*n* = 9 per time point, three cell lines). (C) Volcano plot of D35 vs. D50 proteome. The ECM, matrix metalloproteinase, and CAM proteins from A that are deregulated at D35 vs. D50 secretome are compared with the proteome, and the two ECM proteins with decreased abundance (orange) and two deregulated CAMs (purple) are labeled (FDR<0.05, log_2_fold change values ≤ -1 and ≥1). (D) Heatmap of selected ECM, matrix metalloproteinase, and CAM proteins from A that are present in the proteome. Colors representing the averaged log2 transformed LFQ values at each time point.

Our findings demonstrate that the secretome does not follow a gradual change, and that the secretome is unique at each time point during organoid development. To understand how the secretome changes from D35 to D50, we curated a list of significantly deregulated ECM proteins, matrix metalloproteinases, and cell adhesion molecules from significant GO terms. Then, we visualized the selected proteins on the volcano plot (Figure 4A) and compared their expression at different time points using averaged LFQ values (Figure 4B). Cell adhesion molecules and matrix metalloproteinases were more abundant at D35 compared to the D50, and ECM proteins were more abundant at D50 compared to the D35 in the secretome (Figure 4A), as previously shown in the GO analysis (Figure 3). The heatmap of the selected proteins showed that cell adhesion-related proteins (e.g., NLGN1, NLGN2, NRXN1-3, NRCAM, and L1CAM) clustered together in hierarchical clustering and were enriched at D35 compared to D20 or D50 (Figure 4B). The ECM-related proteins (e.g., Collagen family and Laminin family proteins) were clustered together and enriched at D50 compared to D20 and D35 (Figure 4B). We then validated the presence of the selected CAM and ECM proteins with biologically relevant published proteomic datasets, such as human fetal brain organoid secretome,^12^ human choroid plexus (ChP) organoid-generated CSF,^26^ human embryonic CSF,^26^^,^^27^ human pediatric CSF^26^^,^^28^ and human adult CSF^26^^,^^29^ (Figures S5A and S5B). Most of the selected proteins were also present in published datasets except for neuroligin proteins (Figures S5A and S5B). However, the presence of NLGN1 protein in human adult CSF was shown previously.^30^

Next, to explore whether we would see a similar signature in the organoid proteome, we visualized the same extracellular proteins, as used in Figure 4A, for the D35 and D50 proteome comparison (Figure 4C). We found 22 out of 37 selected proteins were also present in the proteome, but only four were significantly deregulated (Figures 4C and 4D). Additionally, in contrast to the secretome analysis, the hierarchical clustering in the proteome comparison did not show a distinct pattern (Figure 4D). Taken together, secretome at D35 and D50 showed distinct features of extracellular proteins which were not captured by proteomic analysis at the same time points. To understand the similarities and differences between proteome and secretome, we compared the two datasets at different time points (Figures S6A–S6C). The percentage of common proteins between proteome and secretome increased at D35 and D50 (24.2% and 22.6%, respectively) compared to D20 (10.2%) (Figure S6A). GO analysis of common proteins at D20, D35, and D50 included proteins that function in RNA and protein-related processes (Table S4). The distribution of common proteins showed that at D20 and D50, proteins in proteome were clustered at higher LFQ values (Figures S6B and S6C). This suggests a uniform protein expression pattern with relatively high abundance of these proteins in proteome. In contrast, common proteins in secretome were distributed across different LFQ values (Figures S6B and S6C). This could indicate different regulatory mechanisms for secreted proteins than for intracellular proteins. However, at D35, the secreted proteins also clustered at higher LFQ values, a trend that was not observed at other time points (Figures S6B and S6C) and suggests a potential increase in the secretion dynamics at D35. This finding validates the distinct secretion profile of DFOs at D35 compared to D20 and D50 (Figure 3).

Finally, to check for the similarities and differences between proteomics and transcriptomics datasets, we compared the proteome and secretome of DFOs at D50 to the transcriptome of DFOs at D50-55 from a previous study from our lab,^31^ respectively (Figures S7A–S7G). We first identified the common genes/proteins found between proteomic and transcriptomic datasets (Figure S7A), and between the secretome and transcriptomic datasets (Figure S7D). Then, by ranking each individual data point, we calculated the rank difference to assess the similarity between proteome and transcriptome (Figures S7B and S7C), and between secretome and transcriptome (Figures S7E and S7F). We observed that in both comparisons there was a high frequency of genes/proteins that had rank difference close to zero, which indicates a correspondence between protein and RNA levels. The correlation analysis revealed a correlation coefficient of 0.42 between the proteome and transcriptome (Figure S7B), and 0.14 between the secretome and transcriptome (Figure S7E). To identify which genes and proteins might have opposite directionality in the transcriptome and secretome, we selected the 200 genes that had the highest negative difference indicating that they are overrepresented in the secretome and performed GO analysis (Figure S7G). The analysis revealed GO BP terms related to ECM, integrin and cell adhesion were higher in the D50 secretome compared to RNA levels at D50-55 (Figure S7G). This finding emphasizes the importance of secretome analysis in assessing the expression of cell surface and extracellular proteins during neurodevelopment.

## Discussion

In this study, we analyzed the proteome and secretome of human neocortical organoids derived from three control hiPSC lines to understand the developing human neocortex at the protein level. We harvested samples at three developmental time points, aiming to address early time points of neurodevelopment to investigate both different progenitor and neuronal populations and the protein dynamics of neurogenesis. We showed the dynamic composition of the ECM and microenvironment during neural differentiation. While the organoid proteome had signatures of increasing neural differentiation in line with changes in the proportion of progenitor and neuronal populations, the secretome showed a non-linear differentiation trajectory characterized by the increased abundance of synaptic adhesion proteins at D35, when neurogenesis is high.

In line with previous studies,^8^^,^^9^ we confirmed the presence of the main cell types (radial glia cells, IPCs and early born neurons) in the DFOs and characterized their temporal dynamics at D35 and D50 (Figures 1 and S1). We showed that the cells progressed from proliferative toward a more differentiated state over time (Figures 1D and 1E). As cells differentiate, the dynamic expression of temporally patterned genes influences their response to intrinsic and extrinsic cues.^32^ Cells in the later stages of development have increased expression of membrane receptors and cell-cell signaling-related proteins, thus, are also influenced by extrinsic cues.^32^ To investigate the cues that influence DFOs over time, we analyzed the number of secreted proteins at three time points (Figure 3A). We showed that the total number of secreted proteins at D35 and D50 were higher compared to D20. As the neural organoids matured, the cells expressed more cell adhesion molecules and secreted more proteins to their microenvironment and, thus, are possibly influenced by both intrinsic and extrinsic cues.

### Synaptic adhesion molecules, proteases, and CAMs in D35 secretome

**Figure 5:**
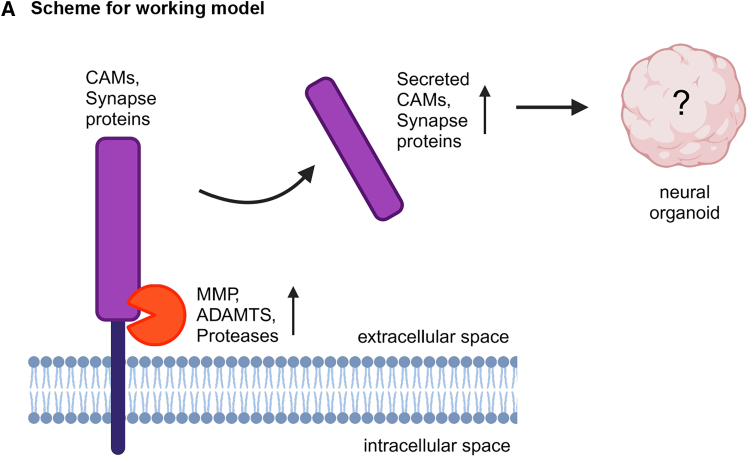
Scheme for the working model (A) An increased abundance of proteases was shown at D35 secretome compared to D50 and D20 (Figures 4A, 4B, and S2C). Proteases can cleave the transmembrane synaptic cell adhesion molecules and secrete them into the extracellular space. This increases the cell adhesion and synapse-related proteins in the secretome (Figure 3G). However, the influence of secreted cell adhesion and synapse-related proteins on neural organoid development is unknown. The working model scheme was created with BioRender.com.

The maturation of neural organoids was accompanied by the gradual increase of synapse-related proteins over time, as shown in the proteome analysis (Figure S4A, S4B and 2G). A previous study showed a gradual increase in the abundance of secreted synapse proteins in the extracellular space upon 2D cortical neuron differentiation.^33^ In contrast to this previous study and to our proteome data, our secretome analysis revealed an increased abundance of synapse-related terms at D35 compared to D50 (Figure 3G). We found secretion of synapse-related neural cell adhesion proteins from the neurexin (NRXN1, NRXN2, and NRXN3) and neuroligin (NLGN1 and NLGN2) families (Figure 4A). Additionally, we observed an increased abundance of proteases, such as MMP15 and ADAMTS7, in D35 secretome compared to D20 and D50 (Figure 4A). Metalloproteinases such as MMP and ADAM/ADAMTS degrade and remodel extracellular proteins.^34^ The increased abundance of these proteases at D35 might be necessary for the dynamic microenvironment needed for proliferating and differentiating cells to co-exist in neural organoids. The extracellular synaptic proteins observed in our secretome data can be secreted from cells via two mechanisms. First, neural-derived extracellular vesicles can carry synapse-related proteins that potentially induce neural differentiation in the neighboring cells,^35^^,^^36^ which has also been shown in neural organoids.^16^ Similar to our secretome data, the content of the extracellular vesicles was shown to vary across different developmental time points, and synapse and cell adhesion-related GO terms were increased in D40 extracellular vesicles.^16^ Second, synaptic proteins can be secreted into the extracellular space via proteases, as we propose in our working model (Figure 5). Proteases can function in ectodomain shedding of synaptic adhesion proteins.^37^ Thus, we propose that secreted synaptic proteins can influence the proliferation or differentiation of neighboring progenitor cells (Figure 5). In previous studies, a similar phenomenon has been shown in glioma cells where neuronal activity-induced cleavage and secretion of transmembrane synaptic protein neuroligin-3 (NLGN3) to the extracellular space promoted proliferation of the neighboring glioma cells.^38^ In conclusion, the secretome analysis highlights a potential importance of synaptic proteins not only in mature neurons but also during early development in guiding the proliferation/differentiation dynamics of the neighboring cells.

Furthermore, cell adhesion molecules are highly significant during neurogenesis. Scuderi et al. showed reduced neurogenesis in monolayer cultures compared to organoids due to the lack of appropriate cell adhesion molecules.^39^ We observed increased secretion of cell adhesion molecules in the D35 secretome compared to D20 and D50 (Figure 4A). Interestingly, the cell adhesion-related protein LGALS3BP was among the proteins with increased abundance in the secretome of D35 organoids. Previously, Kyrousi et al. showed that LGALS3BP was secreted from NPCs in extracellular vesicles and interacted with ECM components to influence corticogenesis at D60.^19^ They also showed that LGALS3BP overexpression at D40 led to a reduced number of SOX2-positive cells and increased neuronal differentiation 4 days later.^19^

### ECM-related changes at D50 secretome

The microenvironment of each cell type at different time points during development is unique, mainly shaped by ECM components such as proteoglycans, laminins, and collagens.^40^ Our analysis revealed increased ECM components in the D50 secretome (Figure 4A), the time point with the highest proportion of neurons (Figure 1D) and an enrichment of synapse-related terms in the D50 proteome (Figure S4B and 2G). ECM modulates neuronal morphology and migration,^40^ and increased ECM density is associated with stabilized neural activity.^41^ Thus, our finding of increased ECM proteins at D50 compared to D35 and D20 suggests an ECM network that mechanically supports differentiated neurons and stabilizes neural connections between them. Hendriks et al. showed ECM rich-basement membrane extract enhanced the maturation of the organoids and these organoids had electrically active neurons at two months as shown by spontaneous and regular calcium spikes.^12^ Together, these results indicate that the composition of the ECM and microenvironment are important factors in neural differentiation, as well as for the formation and maturation of neural networks.

### Microenvironment and neurodevelopmental disorders

Our findings shed light on dynamic changes in the microenvironment in the developing human neocortex at the protein level. The functional importance of such changes is shown in various neurodevelopmental disorders, which may have as a cause ECM disruption during critical developmental stages. For example, disruption in mechanical properties of the microenvironment and ECM stiffness caused by abnormal expression of ECM proteins has been associated with developmental disorders.^42^^,^^43^ In a mouse model of Fragile X syndrome, loss of translational control of matrix metalloproteinase-9 (MMP9) contributed to the disease phenotype, such as delayed dendritic spine maturation.^44^ ECM disruption in perineuronal nets (PNN), neuronal microenvironments that support synapses, has also been associated with Fragile X syndrome, schizophrenia, and epilepsy.^45^ Recently, a human neural organoid model of lissencephaly showed altered stiffening and disorganization of ECM upon LIS1 mutation, which was reverted upon MMP9 treatment.^46^

Interestingly, in more differentiated organoids, one protein that repeatedly ranked among the top five most differentially abundant proteins in the proteome was α-synuclein, which is encoded by the SNCA gene. This protein is highly abundant in presynaptic terminals^47^ and regulates exocytosis and release of neurotransmitters from synaptic vesicles.^48^ Aggregation of α-synuclein protein is associated with Parkinson’s disease and its pathology was previously recapitulated in iPSC-derived neural organoids.^49^^,^^50^ It was also shown that α-synuclein protein can be secreted to the microenvironment, contributing to the disease pathology.^51^ Our data reveals the increased abundance of this neurodegeneration-related protein already at early neurogenesis, suggesting a possible role of neurodegeneration-related proteins in neurodevelopment.

Despite the growing use of organoids in developmental and disease modeling, most studies focus primarily on transcriptomic analysis, with limited insights into protein expression. While the published transcriptomic dataset^31^ captured nearly all expressed and secreted proteins, 0.2% remains unique to either the proteome or secretome and may include functionally significant proteins. This is especially important as organoids are increasingly used as tools to develop drug and cellular targeting systems, where the expression of cell surface receptor molecules and secreted ligands is of high therapeutic interest.

In this study, we provide a comprehensive resource of DFO proteome and secretome dynamics during early development, using three control hiPSC lines. To our knowledge, this is the first study that analyzed the entire secretome of neural organoids at multiple time points. A similar temporal analysis was performed by Pellegrini et al. focusing on cerebrospinal fluid-like secretion from choroid plexus organoids,^26^ and recently by Forero et al. focusing on extracellular vesicles.^16^ Here, we showed the temporal emergence of extracellular proteins and the dynamic microenvironment during brain development. Additionally, we found synaptic proteins highly expressed during neurogenesis indicating roles beyond synapse formation in mature neurons.

### Limitations of the study

To focus on the early neurogenesis stages, we studied the proteome and secretome dynamics of hiPSC-derived DFOs at D20, D35, and D50. However, at these stages, the organoids do not have established neural networks. Therefore, later time points in organoid development should be investigated to study how maturation and functional synaptic connections modulate the proteome and secretome. In this study, we performed proteome and secretome analysis on three male derived-human iPSCs. Although this incorporates ethnic diversity to our dataset, the lack of female-derived iPSC lines may limit the generalizability of our results due to sex-specific differences. Further studies should incorporate both male- and female-derived cell lines to assess sex-specific differences and improve the applicability of the results in a sex-independent manner. We validated the secretome dataset with published datasets from human fetal brain organoids, choroid plexus organoids as well as human CSF samples. Other methods such as western blot or enzyme-linked immunosorbent assay (ELISA) would be beneficial for further validation of proteins of interest. Finally, the proteomic approaches used in this study analyze the proteome and secretome of the organoid in bulk, thereby making cell type-specific inferences, at best, indirect. A recently developed technique, SEC-seq, enables the isolation of single cells and the simultaneous characterization of their secretions and transcriptome.^52^ This technique has not yet been applied to cells of the neural lineage, however, in the future it could be adapted to analyze single-cell secretomes of neural organoids.

## Resource availability

### Lead contact

Further information and requests for resources and reagents should be directed to and will be fulfilled by the Lead Contact, Simone Mayer (simone.mayer@kit.edu).

### Materials availability

This study did not generate new unique reagents or new iPSC cell lines.

### Data and code availability

• Data: All MS data and MS data analysis dataset are submitted and can be accessed at MassIVE: MSV000094878 or ProteomeXchange: PXD052612.
• Code: This study did not generate novel code and the required functions for the analysis and data visualization are described in the STAR Methods section.
• All other items: Further requests to additional information should be directed to the lead contact, Simone Mayer (simone.mayer@kit.edu).

## Acknowledgments

This study was funded by the WIN Program of the 10.13039/100008661Heidelberg Academy of Sciences and Humanities, financed by the Ministry of Sciences, Research, and the Arts of the State of Baden-Württemberg (to SM) and Hertie Foundation (to S.M.). This project was also partially funded by the 10.13039/100000874Brain & Behavior Research Foundation (NARSAD Young Investigator Grant 27026 to S.M.), the Baden-Württemberg state postgraduate fellowship (to K.S. and T.K.), and the Daimler and Benz Foundation (32-06/20, to S.M.). We thank the 10.13039/501100001659German Research Foundation (DFG) for supporting the acquisition of the confocal microscope used in this study (INST 37/1170-1 FUGG, project number 467868227). This study has been partially funded by the 10.13039/501100001659Deutsche Forschungsgemeinschaft (DFG, German Research Foundation) under Germany’s Excellence Strategy via the Excellence Cluster 3D Matter Made to Order (EXC-2082/1 – 390761711).

We thank Elisabeth Gustafsson and Christina Kulka for their technical support. We thank Core Facility for Medical Proteomics, especially Franziska Klose, for protein isolation and mass spectrometry support. We thank Dr. Nicolas Snaidero and his lab members for the confocal microscopy support.

## Author contributions

Z.Y. designed the study, performed experiments, data analysis, statistical analysis, and prepared the manuscript; T.K. and K.S. performed experiments and prepared the manuscript; M.A.J. performed experiments, data analysis, and prepared the manuscript; K.B. performed experiments; all authors revised the manuscript; S.M. conceived and designed the study, supervised the work, and prepared the manuscript.

## Declaration of interests

The authors declare no competing interests.

## STAR★Methods

### Key resources table

**Table:** 

REAGENT or RESOURCE	SOURCE	IDENTIFIER
**Antibodies**		

Goat polyclonal anti-SOX2	R&D Systems	Cat# AF2018; RRID:AB_355110
Rat monoclonal anti-CTIP2	Abcam	Cat# ab18465; RRID:AB_2064130
Rabbit polyclonal anti-Ki67	Millipore	Cat# AB9260; RRID:AB_2142366
Rabbit polyclonal anti-PPP1R17	Atlas Antibodies	Cat# HPA047819; RRID:AB_2680166
Mouse monoclonal anti-HOP	Santa Cruz Biotechnology	Cat# sc-398703; RRID:AB_2687966
Donkey anti-Goat (Alexa Fluor 555)	Abcam	Cat# ab150130; RRID:AB_2927775
Donkey anti-Goat (Alexa Fluor 647)	Invitrogen	Cat# A-21447; RRID:AB_2535864
Donkey anti-Mouse (Alexa Fluor 647)	Invitrogen	Cat# A-31571; RRID:AB_162542
Donkey anti-Rabbit (Alexa Fluor 488)	Invitrogen	Cat# A-21206; RRID:AB_2535792
Donkey anti-Rat (Alexa Fluor 555)	Abcam	Cat# ab150154; RRID:AB_2813834

**Deposited data**		

Mass Spectrometry data of hiPSC-derived DFOs	This paper	MassIVE ID: MSV000094878; ProteomeXchange ID: PXD052612
RNA sequencing data of DFOs	Sarieva et al.^31^	Array Express accession ID: E-MTAB-12702; https://doi.org/10.1038/s41380-023-01997-1
Proteomic dataset of human fetal brain organoid secretome	Hendriks et al.^12^	https://doi.org/10.1016/j.cell.2023.12.012
Proteomic dataset of human choroid plexus (ChP) organoid-generated CSF	Pellegrini et al.^26^	https://doi.org/10.1126/science.aaz5626
Proteomic dataset of human embryonic CSF	Zappaterra et al.^27^	https://doi.org/10.1021/pr070247w
Proteomic dataset of human pediatric CSF	Guo et al.^28^	https://doi.org/10.2147/OTT.S193616
Proteomic dataset of human adult CSF	Dayon et al.^29^	https://doi.org/10.1021/acs.jproteome.8b00809

**Experimental models: Cell lines**		

Human: KOLF2.1J hiPSC	The Jackson Laboratory	hiPSC
Human: BIONi010-C hiPSC	EBiSC	hiPSC
Human: HMGU1 hiPSC	Human Pluripotent Stem Cell Registry	hiPSC

**Software and algorithms**		

BioRender	BioRender	https://www.biorender.com/
ImageJ	Schindelin et al.^56^; Schneider et al.^57^	https://imagej.net/software/fiji/
GraphPad Prism v10	GraphPad Software	https://www.graphpad.com/resources
RStudio v2024.12.0	Posit Software	https://posit.co/downloads/
Perseus suite v1.6.15.0	Perseus Software	https://maxquant.net/perseus/

### Experimental model and study participant details

#### Induced pluripotent stem cell (iPSC) culture

Human male iPSC cell lines KOLF2.1J (Source: The Jackson Laboratory), BIONi010-C (Source: EBiSC), and HMGU1 (Source: Human Pluripotent Stem Cell Registry) were cultured in standard conditions (37°C, 5% CO2, and 100% humidity). BIONi010-C cell lines were cultured in E8 flex medium (Gibco, Cat. no. A2858501), KOLF2.1J and HMGU1 cell line was cultured in mTESR Plus medium (STEMCELL Technologies, Cat. no. 100-0276). All cell lines were tested for mycoplasma contamination using PCR Mycoplasma Detection set (TaKaRa, Cat. no. 6601) and pluripotency using immunocytochemistry with anti-OCT4 antibody (rabbit, 1:500, Abcam, Cat. no. ab19857) upon thawing. For subculturing, Gentle Dissociation Reagent (STEMCELL Technologies, Cat. no. 07174) was used to passage iPSC colonies onto Geltrex-coated (Thermo Fisher Scientific, Cat. no. A1413302) plates. 2 μM Thiazovivin (Sigma, Cat. no. 420220) was added into the respective iPSC culture media for 24 hours after subculturing. Dorsal forebrain organoid differentiation was initiated with iPSCs below passage 20.

#### Dorsal forebrain organoid differentiation

Dorsal forebrain organoids were generated using a previously published protocol with minor modifications.^8^ iPSCs cultures at around 80% confluency were dissociated to single cells using Accutase (MERCK, Cat. no. A6964) and plated in 96 well V-bottom low attachment plates (S-BIO, Cat. no. MS-9096VZ) at 9000 cells per well in specific iPSC media for each cell line. The next day, the aggregate formation was checked, and the media was replaced with cortical differentiation media I (CDMI) supplemented with 20 μM ROCK inhibitor Y-27632 (Cayman Chemical, Cat. no. 10005583), 5 μM SMAD inhibitor SB 431542 (Tocris, Cat. no. 1614), and 3 μM WNT signaling inhibitor IWR-1 (MERCK, Cat. no. 681669). Replacement of iPSC to CDMI media was considered D0 of differentiation. CDMI with all 3 supplements was replaced on D3 of differentiation, and from D6 to D18 of differentiation, CDMI contained only SB and IWR-1 supplements. On D18, dorsal forebrain organoids were transferred to 24-well low-attachment plates and were cultured on an orbital shaker (New Brunswick S41i, 2.5 cm throw, 56 rpm). The media was switched to CDMII from D18 until D35 of differentiation, where media changes were performed every 3-4 days. On D35 of differentiation, the media was switched to CDMIII supplemented with 1% Matrigel (Corning, Cat. no. 356234), and media change was performed twice a week until the organoids were collected for readouts.

### Method details

#### Immunohistochemistry

Immunohistochemistry on DFOs were performed as previously described with minor modifications.^53^ DFOs on the day of readout were fixed with 4% paraformaldehyde in PBS (1x working dilution, Carl Roth, Cat. no. 1105.1) for 1 hour at room temperature (RT). Afterwards, organoids were washed with 1X PBS three times for 15 minutes, then moved to 30% sucrose in PBS solution for at least 2 days at 4°C. Organoids were then embedded in blocks using a 1:1 (v:v) mixture of 30% sucrose and optimal cutting temperature (OCT) compound (TissueTek). The blocks were cryo-sectioned at 20 μm and stored overnight at -20°C before being stored at -80°C for long-term storage.

For immunohistochemistry, the frozen sections were thawed at RT and rehydrated with 1X PBS. Sections were permeabilized and blocked in 1% Triton-X100, 0.2% gelatin, and 10% normal donkey serum in PBS for 1 hour at RT. Next, sections were incubated with primary antibodies overnight at 4°C. The primary antibodies (anti-SOX2 (goat, 1:500, R&D Systems, Cat. no. AF2018), anti-CTIP2 (rat, 1:500, Abcam, Cat. no. ab18465), anti-Ki67 (rabbit, 1:600, Merck, Cat. no. AB9260), anti-PPP1R17 (rabbit, 1:1000, Atlas Antibodies, HPA047819) and anti-HOPX (mouse, 1:250, Santa Cruz, Cat. no. sc-398703)) were diluted in permeabilization and blocking solution. The next day, the sections were washed three times with 1X PBS for 15 minutes, and secondary antibody incubation was performed for 3 hours at RT protected from light. The secondary antibodies (1:1000, Table S1) were diluted in permeabilization and blocking solution. Afterwards, sections were washed three times with 1X PBS for 15 minutes and stained with 1:5000 nuclear dye DAPI (Thermofisher Scientific, Cat. no. D1306) in PBS for 5 minutes. After the final wash with 1X PBS, the organoid sections were mounted with ProLong™ Gold Antifade Mountant (Thermo Fisher Scientific, Cat. no. P36930). Images were then acquired with an Olympus FV3000 confocal microscope using a 20x magnification objective. Three randomly selected VZ-like regions were imaged per organoid for quantification. In cases when organoids had less than three or no VZ-like regions, organoid edges with SOX2-positive areas were randomly imaged to make a total of three regions of interest per organoid.

#### Sample collection and protein isolation

Whole organoid proteome and secretome samples were collected as organoids or their culturing media, respectively, from the same organoids. Within each sample, 3 organoids or 3 media aliquots from respective organoids were pooled. For secretome analysis, media change was performed on D19, D34, and D49 to collect one-day old media on D20, D35, and D50. Media were collected into a 2 mL tube, submerged in liquid nitrogen until frozen, and stored at -80°C. For D49 media change, CDMIII media without HyClone Defined Fetal Bovine Serum (Cytiva, Cat. no. SH30070.03) and Matrigel was used to avoid artifacts in protein composition. For whole organoid proteome analysis, after collection of the culturing media, dorsal forebrain organoids on D20, D35, and D50 of differentiation were washed with 1X PBS, then transferred to protein LoBind 1.5 mL tubes (Eppendorf, Cat. no. 0030108116). Once transferred, any liquid was removed, and tubes containing organoids were submerged in liquid nitrogen for snap freezing and stored at -80°C until protein isolation. The samples were delivered to the Core Facility for Medical Bioanalytics for protein isolation and mass spectrometry.

Total protein was extracted from collected samples using TBS lysis buffer (Tris-(hydroxymethyl)-aminomethane (30mM; Tris (AppliChem), NP40 0.5%, supplemented with complete protease inhibitors cocktail (Roche, Cat. no. 11836170001) and phosphatase inhibitors 2 and 3 cocktails (Sigma Cat. no. P5726 and Cat. no. P004) according to the manufacturer recommendations. Total extracted proteins were precipitated and quantified using Bradford assay, and a similar total amount of protein from each sample was used to prepare the sample for mass spectrometry analysis as described previously.^54^

For secretome, an equal amount of secretome collected from different biological conditions were centrifuged at 5,000 g for 10 minutes at 4°C to remove cell debris and subjected to a methanol/chloroform precipitation as adapted from a published study.^55^ Briefly, the collected supernatant volume was mixed with four volumes ice-cold methanol, vortexed and centrifuged for one minute at 9,000 g. Then, mixed with one volume of chloroform, vortexed and centrifuged for one minute at 9,000 g. Three volumes of HPLC grade water were added, vortexed and centrifuged for two minutes at 16,000 g. After discarding the upper phase, without disturbing the interphase, three volumes of HPLC grade water were added, vortexed and centrifuged at 16,000 g for four minutes. Finally, the supernatant was discarded, and the protein pellets were dried under a laminar flow hood and stored at -80°C to be processed for in solution tryptic digestion.

Briefly, extracted proteins were alkylated and reduced using dithiothreitol (DTT) and iodoacetamide (IAA), followed by tryptic digestion overnight at 4°C. Collected peptides were further desalted using STAGE-tip (Affinisep). Peptides were resuspended in Acetonitrile trifluoracetic acid solution and prepared for Mass spectrometry analysis.

#### Mass spectrometry (MS)

Mass Spectrometry analysis was performed on an Ultimate3000 RSLC system coupled to an Orbitrap Tribrid Fusion mass spectrometer (Thermo Fisher Scientific). Tryptic peptides were loaded onto a μPAC Trapping Column with a pillar diameter of 5 μm, inter-pillar distance of 2.5 μm, pillar length/bed depth of 18 μm, external porosity of 9%, bed channel width of 2 mm and length of 10 mm; pillars are superficially porous with a porous shell thickness of 300 nm and pore sizes in the order of 100 to 200 Å at a flow rate of 10 μl per min in 0.1% trifluoroacetic acid in HPLC-grade water. Peptides were eluted and separated on the PharmaFluidics μPAC nano-LC column: 50 cm μPAC C18 with a pillar diameter of 5 μm, inter-pillar distance of 2.5 μm, pillar length/bed depth of 18 μm, external porosity of 59%, bed channel width of 315 μm and bed length of 50 cm; pillars are superficially porous with a porous shell thickness of 300 nm and pore sizes in the order of 100 to 200 Å by a linear gradient from 2% to 30 % of buffer B (80% acetonitrile and 0.08% formic acid in HPLC-grade water) in buffer A (2% acetonitrile and 0.1% formic acid in HPLC-grade water) at a flow rate of 300 nl per min. The remaining peptides were eluted by a short gradient from 30% to 95% buffer B; the total gradient run was 120 min. Spectra were acquired in Data Independent Acquisition (DIA) mode using 50 variable-width windows over the mass range 350-1500 m/z, MS2 scan range was set from 200 to 2000 m/z.

### Quantification and statistical analysis

#### Image preprocessing and analysis

For quantification of SOX2, CTIP2, and PPP1R17 positive cell types, a column with a fixed 100 μm width was cropped from the whole image using the lumen of a VZ-like region as the upper limit and the edge of the organoid as the lower limit. For organoid sections without VZ-like regions, SOX2+ cells were used as a guidance to select the region of interest (to be comparable to VZ-like regions), then a column with a fixed 100 μm width was randomly cropped from the whole image using the edge of the organoid section as the lower limit, similar to quantification of VZ-like regions. To quantify proliferative cells within VZ-like regions, VZ-like regions were cropped based on the expression of SOX2 vRG marker. Prior to image analysis, to blind the quantification process, all images within the exact cell type quantification (including D35 and D50 with and without VZ) were collected in a folder where images were randomly assigned numbers using a custom R code.

After randomization, images were analyzed using ImageJ software^56^^,^^57^ and a custom ImageJ macro. In short, the custom ImageJ macro first selects and measures the area positive for the normalization channel using a manually selected threshold. Then, within the previously selected area measure the area positive for the channel of interest using a manually selected threshold for each image. For SOX2 and CTIP2 staining, the custom ImageJ macro first measured the DAPI-positive area and then the SOX2- or CTIP2-positive area within the DAPI-positive area. For cytosolic markers of intermediate progenitor cells PPP1R17, the PPP1R17-positive area was measured and normalized to the total area of the region of interest. Finally, for proliferative vRG, the area positive with the SOX2 marker and the area positive with the Ki-67 marker within the SOX2-positive area were measured. At the end of quantification, randomized images were assigned back to their original image names in Microsoft Excel, and the channel of interest area/normalization channel area was calculated. The data was plotted using GraphPad Prism 9.

#### Quantification statistics

The statistics on the image quantifications were performed in RStudio software using custom R scripts. First, the data was checked for normality using the Shapiro-Wilk normality test. Most of the data was not normally distributed. Therefore, ARTool package and TukeyHSD() function were used for a non-parametric Aligned Rank Transform (ART) ANOVA test and Tukey's test for multiple comparisons, respectively. The day variable was used as an independent variable, and the cell line variable was included as a covariate for the ART ANOVA test. Tukey's test was only performed on the day variable. The details of the statistical test results, exact value of n, what n represents, definition of center and dispersion and precision measures can be found in the respective figure legends.

#### MS data analysis and statistics

MS RAW data were analyzed using DIA-NN 1.8.1^58^ in library-free mode against the human database (UniProt release September 2023). First, a precursor ion library was generated using FASTA digest for library-free search in combination with deep learning-based Spectra prediction. An experimental library generated from the DIA-NN search was used for cross-run normalization and Mass accuracy correction. Only high-accuracy spectra with a minimum precursor false discovery rate (FDR) of 0.01, and only tryptic peptides (2 missed Tryptic cleavages) were used for protein quantification. The match between runs option was activated and no shared spectra were used for protein identification.

Statistical analysis, including label-free quantification ratios (LFQ), and two-sided corrected permutation-based T-test (250 permutations and a minimum p-value of 0.05) to identify putative differentially abundant proteins between the two groups was done using the Perseus software suite version 1.6.15.0.^59^ The analytical platform Omics Playground (https://github.com/bigomics/omicsplayground) was used for proteomics data exploration and integration.

Principle component analysis (PCA) plots and volcano plots were generated using the built-in functions within the Perseus software. For PCA and Venn diagrams, data before data imputation was used, and this information can be found in the figure captions. For the rest of the analysis, data imputation was performed separately for each cell line and time point by replacing missing values with values from normal distribution. Custom codes in RStudio software were generated for Venn diagrams, heatmaps, GO analysis, scatter plots, density plots and histograms. To analyze and visualize proteins, we used gene symbols. During the protein inference procedure, due to the shared peptides among protein groups, multiple or repeating gene symbols were observed. In the case of multiple gene symbols, we kept only the first symbol; in the case of multiple appearances of the same gene symbol (repeating gene symbols), depending on the analysis, either one or all were used unless otherwise noted in the figure legend. ggvenn library was used for the generation of Venn diagrams and extraction of distinct/common proteins that were used in further analysis. Repeating gene symbols were used once in the Venn diagrams. pheatmap library was used for heatmaps with “Euclidean” as clustering distance for both rows and columns and averaged log2 transformed LFQ values were used as input for the heatmaps. To compare the proteomics data to a previously published transcriptomics dataset,^31^ we used the averaged raw gene counts from the transcriptomics data and averaged LFQ values from the proteomics data. Rank of the averaged LFQ values and rank of the averaged raw gene counts were used to visualize the heatmap plots without any clustering. pheatmap function was also used for checking the presence of selected CAM and ECM proteins in published proteomic datasets: human fetal brain organoid secretome,^12^ human choroid plexus organoid-produced CSF,^26^ human embryonic CSF,^26^^,^^27^ human pediatric CSF^26^^,^^28^ and human adult CSF.^26^^,^^29^ The presence of the selected protein was displayed with a colored box for the given dataset. Density plots, scatter plots and histograms were generated using ggplot function. For histogram visualizing the relationship between proteome/secretome and transcriptome, rank difference between averaged LFQ values and averaged gene counts was used. Correlation test between datasets was performed using Pearson test in RStudio software. For scatter plots and density plots comparing proteome and secretome, averaged log10 transformed LFQ values of the common proteins that were found in both proteome and secretome were used for each respective day. Overrepresentation analysis (ORA) for GO terms was performed using topGO library and parameters used for enrichGO function were as follows: reference gene list=org.Hs.eg.db, minGSSize=10, maxGSSize=500, p value=0.05, q value=0.10 and p adjusted method = FDR. Further adjustments to the plots were made using Inkscape software. Polar plots comparing proteome and secretome of three cell lines were generated by a custom code in RStudio software using volcano3D package.^60^ In short, the code calculated the mean LFQ values for each cell line for the given time point and map the information onto polar coordinates along three-axis.

## Supplemental information

Document S1. Figures S1–S7 and Table S1

Table S2. Excel file containing mass spectrometry data from DFO proteome and secretome before data imputation

Table S3. Excel file containing mass spectrometry data from DFO proteome and secretome after data imputation

Table S4. Excel file containing mass spectrometry data from DFO proteome and secretome comparison at D20, D35 and D50
