# Supplementary material for: Human dorsal forebrain organoids show differentiation-state-specific protein secretion
Source: iScience. 2025 Jun 19;28(7):112935. doi: 10.1016/j.isci.2025.112935 (PMC12270659; doi:10.1016/j.isci.2025.112935)
Supplement: Document S1. Figures S1–S7 and Table S1 [file mmc1.pdf]

iScience, Volume *28*

Supplemental information

Human dorsal forebrain organoids show differentiation-state-specific protein secretion

Zeynep Yentür, Theresa Kagermeier, Kseniia Sarieva, Mohamed A. Jarboui, Katharina Becker, and Simone Mayer

Figure S1

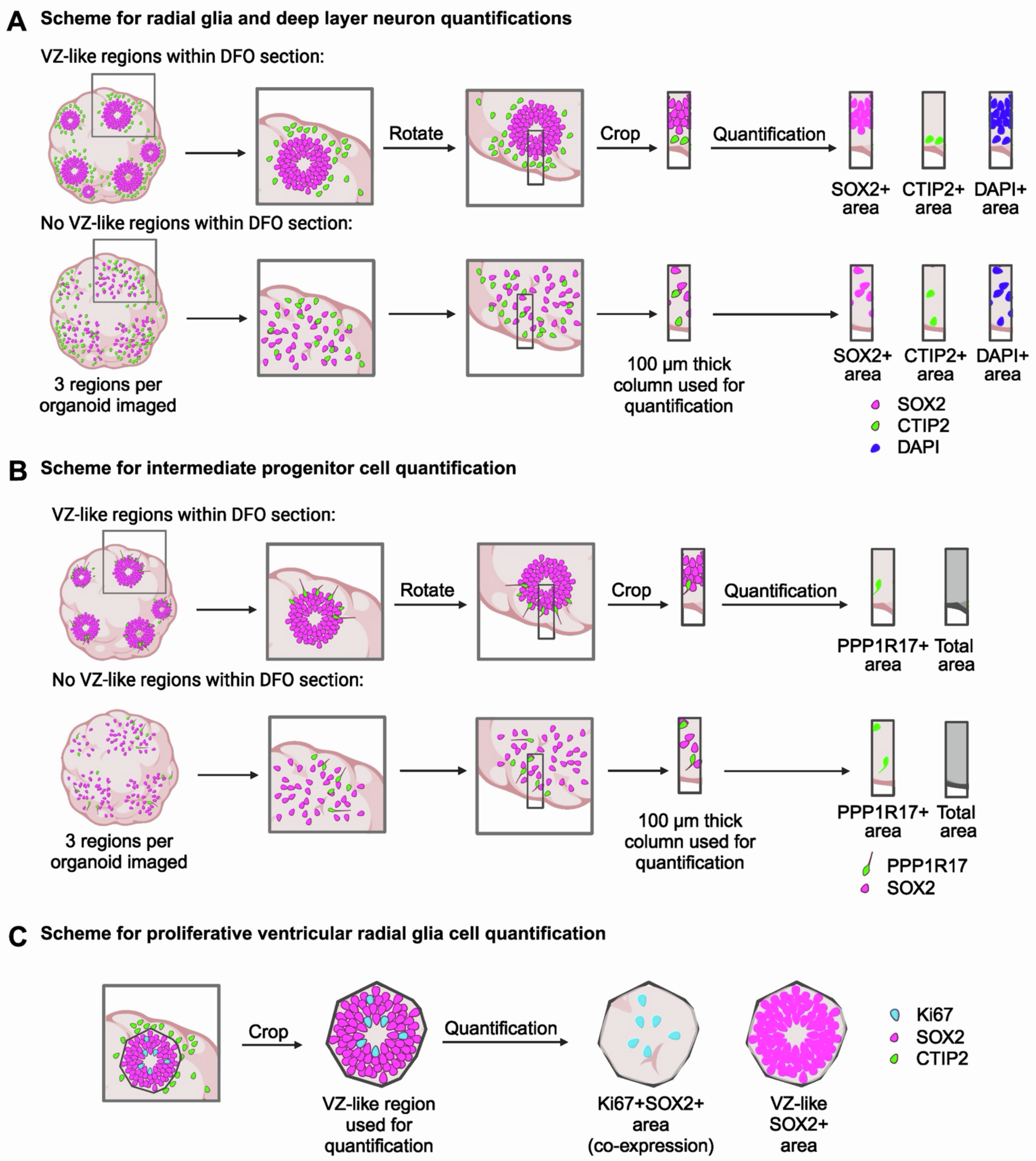

A Scheme for radial glia and deep layer neuron quantifications
VZ-like regions within DFO section:
Rotate
Crop
Quantification
SOX2+ area
CTIP2+ area
DAPI+ area
No VZ-like regions within DFO section:
3 regions per organoid imaged
100 µm thick column used for quantification
SOX2+ area
CTIP2+ area
DAPI+ area
SOX2
CTIP2
DAPI
B Scheme for intermediate progenitor cell quantification
VZ-like regions within DFO section:
Rotate
Crop
Quantification
PPP1R17+ area
Total area
No VZ-like regions within DFO section:
3 regions per organoid imaged
100 µm thick column used for quantification
PPP1R17+ area
Total area
PPP1R17
SOX2
C Scheme for proliferative ventricular radial glia cell quantification
Crop
Quantification
VZ-like region used for quantification
Ki67+SOX2+ area (co-expression)
VZ-like SOX2+ area
Ki67
SOX2
CTIP2

Figure S1: Scheme for cell type quantifications.

A Scheme for radial glia and early born neuron quantification. A 100 µm thick column was cropped for cell-type quantifications in organoids with VZ-like regions. The whole frame at the edge of the organoid was imaged for organoids with no VZ-like region. A 100 µm thick column was cropped from the edge of the organoid. Three columns per organoid were quantified using cell marker (SOX2 or CTIP2) positive area over DAPI positive area.

B Scheme of isolation of VZ-like regions using SOX2 staining. VZ-like regions per organoid were quantified using SOX2 and Ki67 double positive area over SOX2 positive area.

C Scheme for intermediate progenitor quantification. Columns were prepared similarly to **A**, three columns per organoid were quantified using cell marker PPP1R17 positive area over the total organoid area within the column.

Illustrations for cell quantification schemes in **A, B,** and **C** were created with BioRender.com.

Figure S2

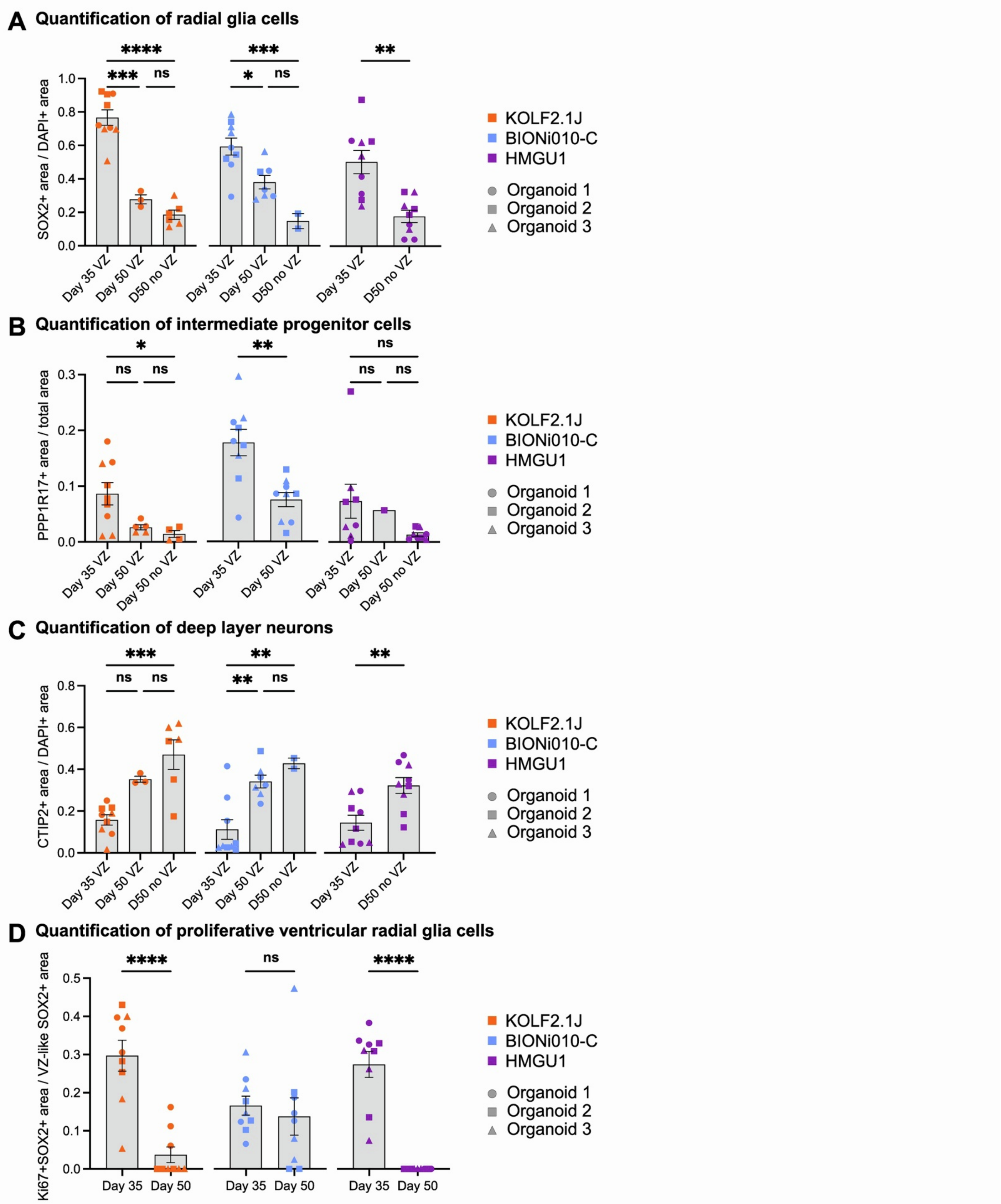
A Quantification of radial glia cells
SOX2+ area / DAPI+ area
Day 35 VZ
Day 50 VZ
D50 no VZ
B Quantification of intermediate progenitor cells
PPP1R17+ area / total area
Day 50 no VZ
C Quantification of deep layer neurons
CTIP2+ area / DAPI+ area
D Quantification of proliferative ventricular radial glia cells
Ki67+SOX2+ area / VZ-like SOX2+ area
Day 35
Day 50
KOLF2.1J
BIONi010-C
HMGU1
Organoid 1
Organoid 2
Organoid 3

Figure S2: Cell type quantifications at D35 and D50 split by cell lines.

A Quantification of SOX2 signal area over DAPI signal area split by cell lines KOLF2.1J, BIONi010-C and HMGU1. D35 VZ (n=9), D50 VZ (n=3), and D50 no VZ (n=6) for KOLF2.1J. D35 VZ (n=9), D50 VZ (n=7), and D50 no VZ (n=2) for BIONi010-C. D35 VZ (n=9), and D50 no VZ (n=9) for HMGU1.

B Quantification of PPP1R17 positive intermediate progenitor area over total area split by cell lines KOLF2.1J, BIONi010-C and HMGU1. D35 VZ (n=9), D50 VZ (n=5), and D50 no VZ (n=4) for KOLF2.1J. D35 VZ (n=9), and D50 VZ (n=9) for BIONi010-C. D35 VZ (n=8), D50 VZ (n=1), and D50 no VZ (n=8) for HMGU1.

C Quantification of CTIP2 signal area over DAPI signal area split by cell lines KOLF2.1J, BIONi010-C and HMGU1. D35 VZ (n=9), D50 VZ (n=3), and D50 no VZ (n=6) for KOLF2.1J. D35 VZ (n=9), D50 VZ (n=7), and D50 no VZ (n=2) for BIONi010-C. D35 VZ (n=9), and D50 no VZ (n=9) for HMGU1.

D Quantification of Ki67 and SOX2 double positive proliferative ventricular radial glia cells (vRGs) split by cell lines KOLF2.1J, BIONi010-C and HMGU1. D35 VZ (n=9) and D50 (n=9) for all cell lines. Organoids that do not have VZ-like regions were plotted and analyzed as 0.

For quantification panels **A** - **D**, bars represent the mean of each quantification, and error bars represent ± SEM. For all, Aligned Rank Transform (ART) ANOVA was used to compare the conditions as some of the data failed the normality test. Tukey´s test was performed for multiple comparisons between day conditions. Data points represent individual quantifications. The color of the data points represents cell line, KOLF2-1J, orange; BIONi010-C, green; HMGU1, purple, one batch per cell line was quantified. The shape of the data points represents individual organoids, three organoids per cell line were quantified. ns, non-significant p-value > 0.05; * p-value < 0.05; ** p-value < 0.01; *** p-value < 0.001; **** p-value < 0.0001.

Figure S3

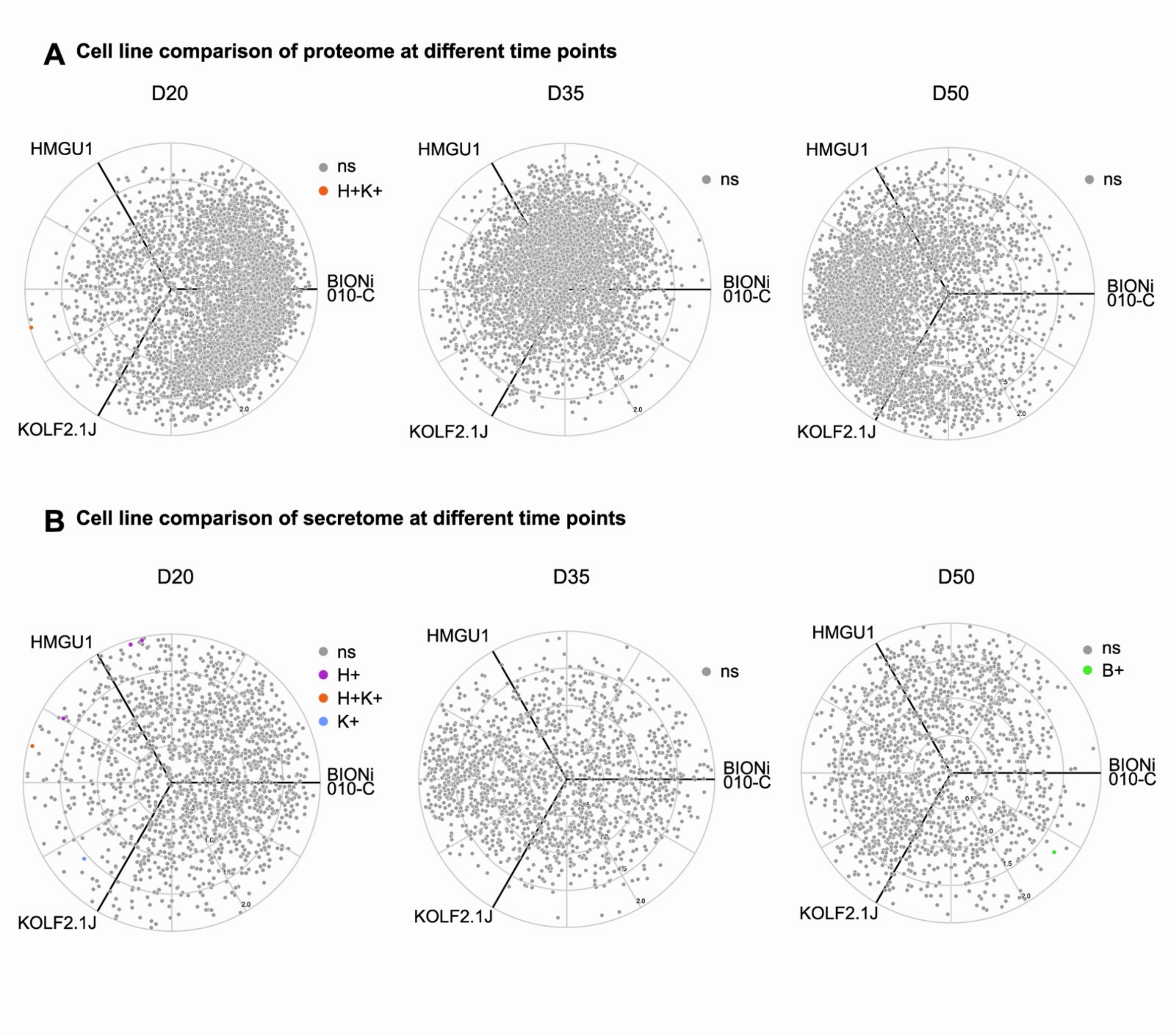
A Cell line comparison of proteome at different time points
D20
D35
D50
HMGU1
BIONi 010-C
KOLF2.1J
ns
H+K+
B Cell line comparison of secretome at different time points
D20
D35
D50
HMGU1
BIONi 010-C
KOLF2.1J
ns
H+
H+K+
K+
B+

Figure S3: Cell line comparison of proteome and secretome at different time points

A Differentially abundant proteins in proteome at D20 (left), D35 (middle) and D50 (right) comparing KOLF2.1J, BIONi010-C and HMGU1 cell lines. Each dot represents a protein, significant proteins are color-coded, and non-significant proteins are in grey. One protein was significantly abundant at D20 for HMGU1+KOLF2.1J (in orange).

B Differentially abundant proteins in secretome at D20 (left), D35 (middle) and D50 (right) comparing KOLF2.1J, BIONi010-C and HMGU1 cell lines. Each dot represents a protein, significant proteins are color-coded, and non-significant proteins are in grey. Five proteins were differentially abundant at D20, three proteins for HMGU1 (in purple), one protein for HMGU1+KOLF2.1J (in orange) and one protein for KOLF2.1J (in blue). One protein was significantly abundant at D50 for BIONi010-C (in green).

Figure S4

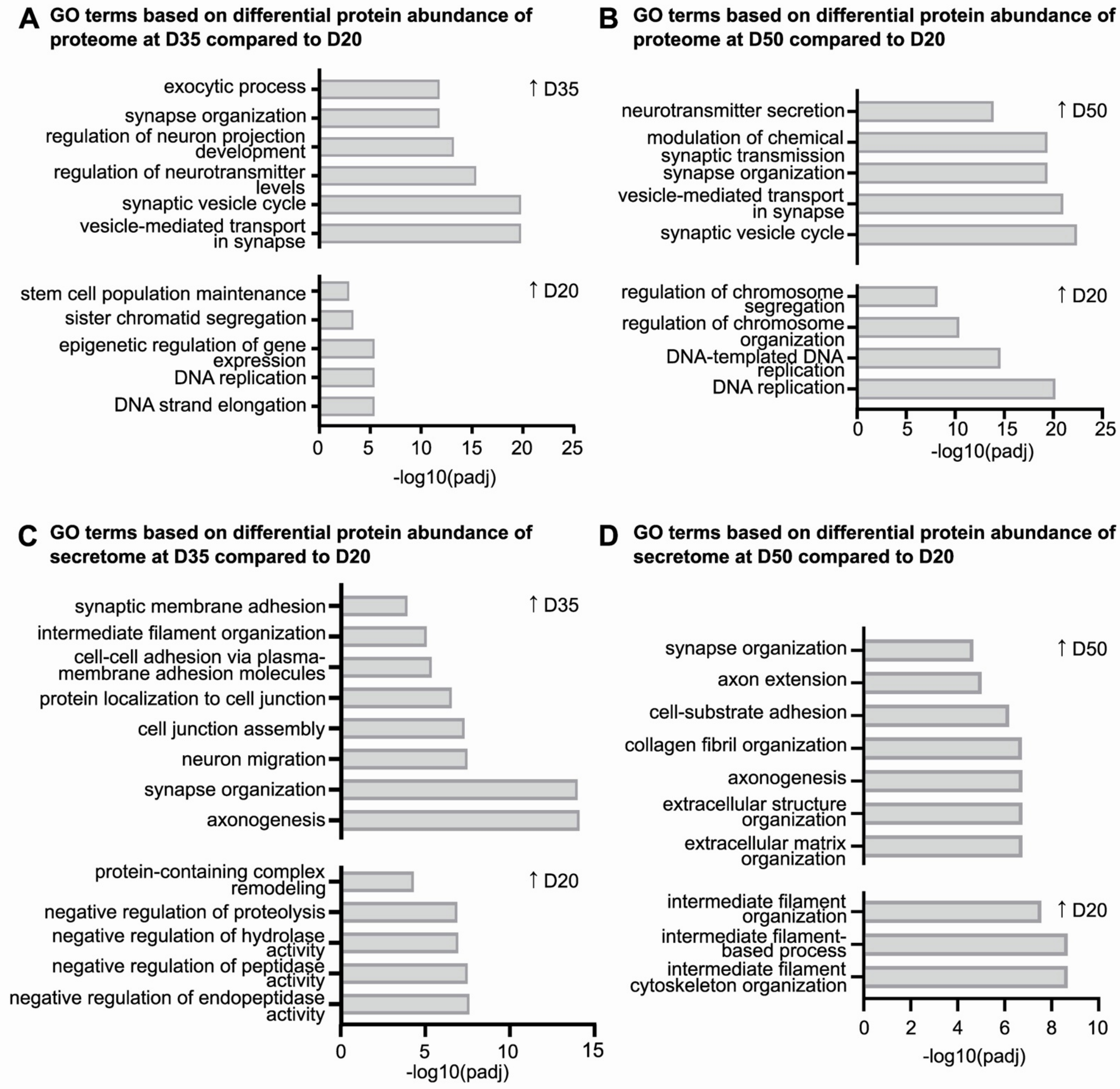
A GO terms based on differential protein abundance of proteome at D35 compared to D20
↑ D35
exocytic process
synapse organization
regulation of neuron projection development
regulation of neurotransmitter levels
synaptic vesicle cycle
vesicle-mediated transport in synapse
↑ D20
stem cell population maintenance
sister chromatid segregation
epigenetic regulation of gene expression
DNA replication
DNA strand elongation
0 5 10 15 20 25
-log10(padj)
B GO terms based on differential protein abundance of proteome at D50 compared to D20
↑ D50
neurotransmitter secretion
modulation of chemical synaptic transmission
synapse organization
vesicle-mediated transport in synapse
synaptic vesicle cycle
↑ D20
regulation of chromosome segregation
regulation of chromosome organization
DNA-templated DNA replication
DNA replication
0 5 10 15 20 25
-log10(padj)
C GO terms based on differential protein abundance of secretome at D35 compared to D20
↑ D35
synaptic membrane adhesion
intermediate filament organization
cell-cell adhesion via plasma-membrane adhesion molecules
protein localization to cell junction
cell junction assembly
neuron migration
synapse organization
axonogenesis
↑ D20
protein-containing complex remodeling
negative regulation of proteolysis
negative regulation of hydrolase activity
negative regulation of peptidase activity
negative regulation of endopeptidase activity
0 5 10 15
-log10(padj)
D GO terms based on differential protein abundance of secretome at D50 compared to D20
↑ D50
synapse organization
axon extension
cell-substrate adhesion
collagen fibril organization
axonogenesis
extracellular structure organization
extracellular matrix organization
↑ D20
intermediate filament organization
intermediate filament-based process
intermediate filament cytoskeleton organization
0 2 4 6 8 10
-log10(padj)

Figure S4: Gene Ontology (GO) biological processes terms for proteome and secretome at different time points.

A GO Biological processes analysis of proteins with increased (top) and decreased (bottom) abundance in the organoid proteome at D35 vs. D20 from **Fig. 2D**.

B GO Biological processes analysis of proteins with increased (top) and decreased (bottom) abundance in the organoid proteome at D50 vs. D20 from **Fig. 2E**.

C GO biological processes analysis of proteins with increased (top) and decreased (bottom) abundance from D35 vs. D20 secretome analysis from **Fig. 3D**.

D GO biological processes analysis of proteins with increased (top) and decreased (bottom) abundance from D50 vs. D20 secretome analysis from **Fig. 3E**.

Figure S5

A Presence of selected CAM proteins in human fetal brain organoid secretome, human ChP organoid-produced CSF and human CSF from published datasets

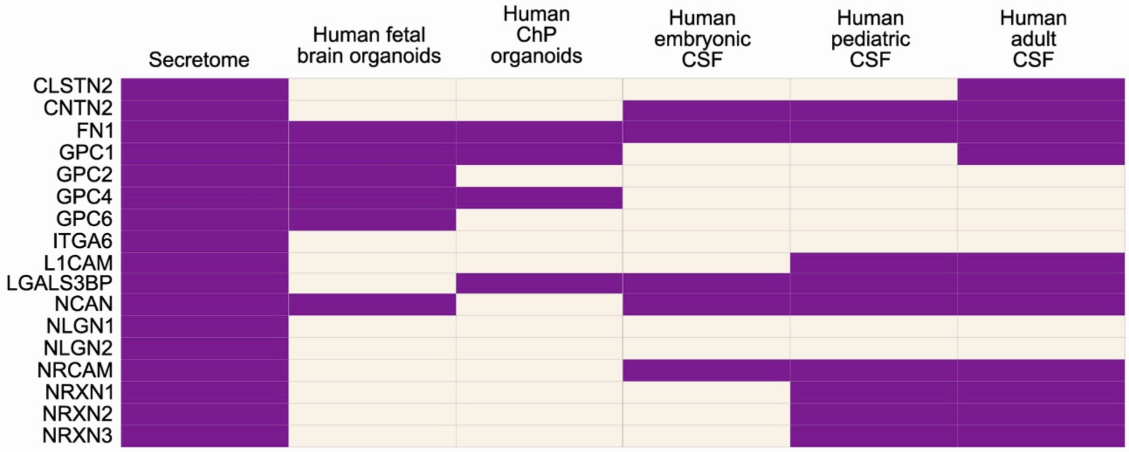

B Presence of selected ECM proteins in human fetal brain organoid secretome, human ChP organoid-produced CSF and human CSF from published datasets

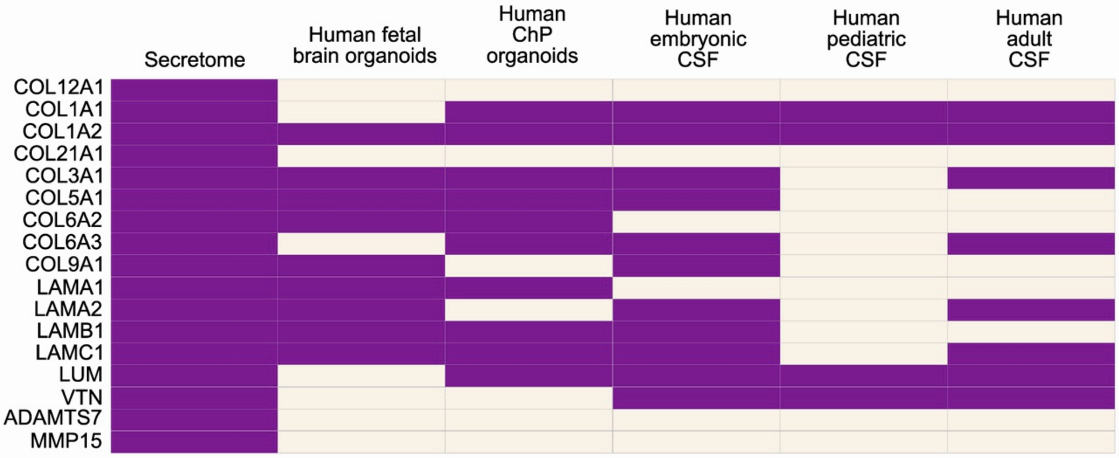

Figure S5: Presence of selected CAM and ECM proteins in other published datasets.

A Presence of selected CAM proteins from **Fig. 4B** in published proteomics datasets from human fetal organoid secretome, choroid plexus organoid-produced CSF, human embryonic CSF, human pediatric CSF and human adult CSF. Purple colored boxes indicate the presence of the protein in the respective dataset.

B Presence of selected ECM and protease proteins from **Fig. 4B** in published proteomics datasets from human fetal brain organoid secretome[1], choroid plexus organoid-produced CSF[2], human embryonic CSF[3], human pediatric CSF[4] and human adult CSF[5]. Purple colored boxes indicate the presence of the protein in the respective dataset.

Figure S6

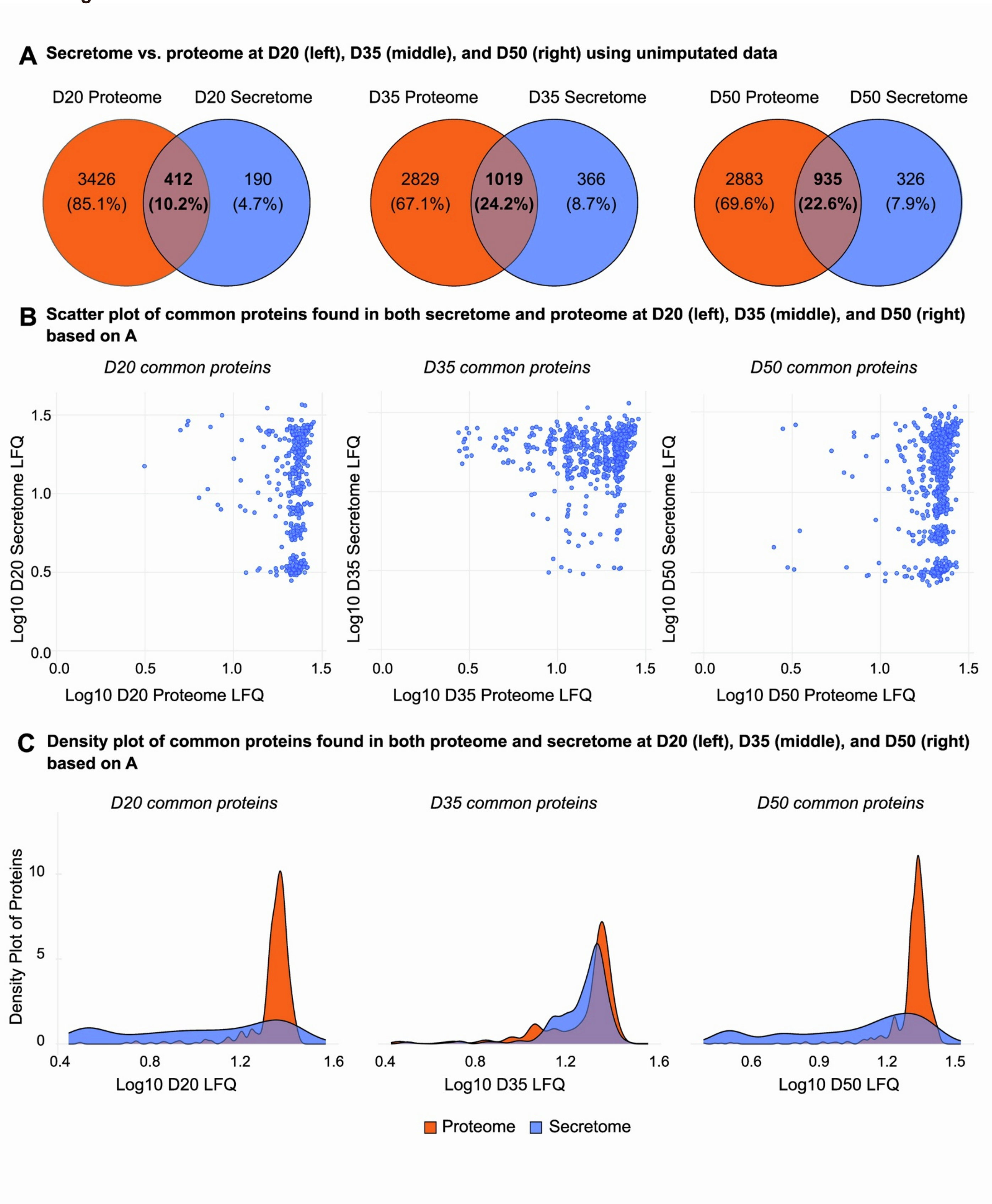
A Secretome vs. proteome at D20 (left), D35 (middle), and D50 (right) using unimputated data
D20 Proteome
D20 Secretome
3426 (85.1%)
412 (10.2%)
190 (4.7%)
D35 Proteome
D35 Secretome
2829 (67.1%)
1019 (24.2%)
366 (8.7%)
D50 Proteome
D50 Secretome
2883 (69.6%)
935 (22.6%)
326 (7.9%)
B Scatter plot of common proteins found in both secretome and proteome at D20 (left), D35 (middle), and D50 (right) based on A
D20 common proteins
D35 common proteins
D50 common proteins
Log10 D20 Secretome LFQ
Log10 D35 Secretome LFQ
Log10 D50 Secretome LFQ
Log10 D20 Proteome LFQ
Log10 D35 Proteome LFQ
Log10 D50 Proteome LFQ
C Density plot of common proteins found in both proteome and secretome at D20 (left), D35 (middle), and D50 (right) based on A
D20 common proteins
D35 common proteins
D50 common proteins
Density Plot of Proteins
Log10 D20 LFQ
Log10 D35 LFQ
Log10 D50 LFQ
Proteome
Secretome

Figure S6: Comparison of proteome and secretome datasets at different time points.

A Venn diagram of proteins found in the organoid proteome and secretome at D20 (left), D35 (middle) and D50 (right). Proteins found in at least one sample were included for both proteome and secretome and values before data imputation were used. Repeating proteins were counted once.

B Scatter plot of proteins found common in both proteome and secretome at D20 (left), D35 (middle) and D50 (right). Log10 of LFQ values were calculated from unimputated data and plotted against proteome vs. secretome. Each dot represents a protein that is found in the intersection of **A**.

C Density plot of proteins found common in both proteome and secretome at D20 (left), D35 (middle) and D50 (right). Log10 of LFQ values from unimputated data were used. Density of proteins found in proteome were plotted in orange, while the density of proteins found in secretome were plotted in blue.

For **B** and **C**, the first occurrence of the protein was kept for the analysis.

Figure S7

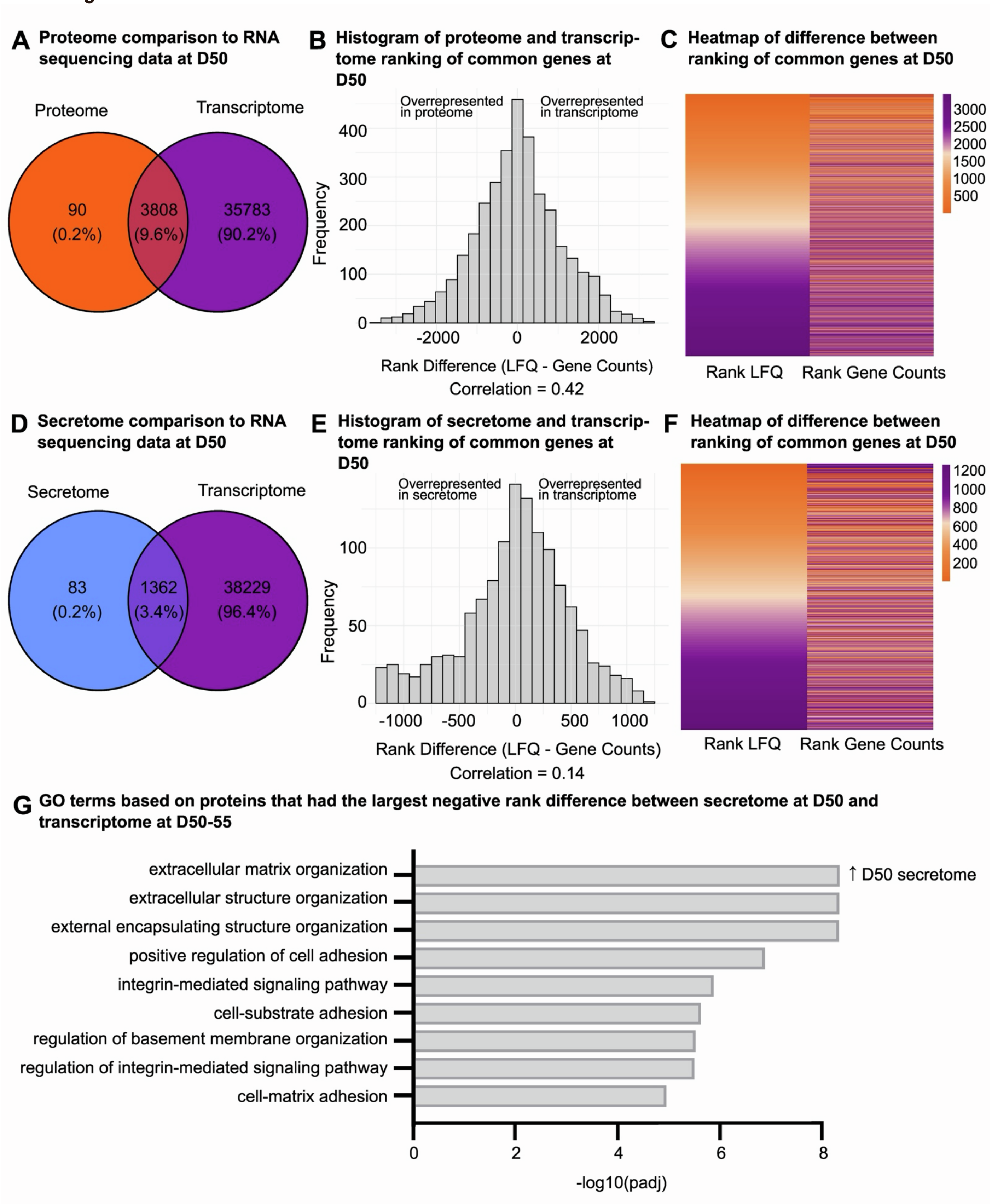
A Proteome comparison to RNA sequencing data at D50
Proteome
Transcriptome
90
(0.2%)
3808
(9.6%)
35783
(90.2%)
B Histogram of proteome and transcriptome ranking of common genes at D50
Overrepresented in proteome
Overrepresented in transcriptome
Frequency
Rank Difference (LFQ - Gene Counts)
Correlation = 0.42
C Heatmap of difference between ranking of common genes at D50
Rank LFQ
Rank Gene Counts
D Secretome comparison to RNA sequencing data at D50
Secretome
Transcriptome
83
(0.2%)
1362
(3.4%)
38229
(96.4%)
E Histogram of secretome and transcriptome ranking of common genes at D50
Overrepresented in secretome
Overrepresented in transcriptome
Frequency
Rank Difference (LFQ - Gene Counts)
Correlation = 0.14
F Heatmap of difference between ranking of common genes at D50
Rank LFQ
Rank Gene Counts
G GO terms based on proteins that had the largest negative rank difference between secretome at D50 and transcriptome at D50-55
extracellular matrix organization
extracellular structure organization
external encapsulating structure organization
positive regulation of cell adhesion
integrin-mediated signaling pathway
cell-substrate adhesion
regulation of basement membrane organization
regulation of integrin-mediated signaling pathway
cell-matrix adhesion
↑ D50 secretome
-log10(padj)

Figure S7: Comparison of proteome and secretome datasets at D50 to published transcriptome dataset at D50-55.

A Venn diagram of proteins found in the organoid proteome at D50 compared to genes found in organoid transcriptome at D50-55[6]. Transcriptome dataset includes all genes, including non-protein coding genes and protein coding genes. Repeating proteins/genes were counted once.

B Histogram of proteins/genes found common in both proteome and transcriptome datasets. The common genes were individually ranked for proteome and transcriptome using LFQ values for proteome and gene counts for transcriptome. Difference between the ranks (LFQ rank – Gene Counts rank) were used to plot the histogram.

C Heatmap of proteins/genes found common in both proteome and transcriptome datasets. Ranked values of LFQ from proteome dataset and ranked values of Gene Counts from transcriptome dataset were used.

D Venn diagram of proteins found in the organoid secretome at D50 compared to genes found in organoid transcriptome at D50-55. Repeating proteins were counted once.

E Histogram of proteins/genes found common in both secretome and transcriptome datasets. The common genes were individually ranked for secretome and transcriptome using LFQ values for proteome and gene counts for transcriptome. Difference between the ranks (LFQ rank – Gene Counts rank) were used to plot the histogram. The negative difference values represent proteins with high LFQ values but low gene counts.

F Heatmap of proteins/genes found common in both secretome and transcriptome datasets. Ranked values of LFQ from secretome dataset and ranked values of Gene Counts from transcriptome dataset were used. The heatmap was organized according to the LFQ ranks, in which top proteins exhibit the lowest LFQ rank (indicated by orange) while having the highest gene count ranks (indicated by purple).

G GO Biological processes analysis of proteins with highest 200 negative difference value (higher LFQ rank compared to Gene Counts rank) from **E**.

For **B**, **C**, **E** and **F**, repeating proteins/genes were not included in the analysis.

Table S1. Secondary antibodies, related to STAR Methods

Host species	Target species	Fluorophore	Provider	Cat. no
Donkey	Goat	555	Abcam	ab150130
Donkey	Goat	647	Invitrogen	A21447
Donkey	Mouse	647	Invitrogen	A31571
Donkey	Rabbit	488	Invitrogen	A21206
Donkey	Rat	555	Abcam	ab150154

References

1. Hendriks, D., Pagliaro, A., Andreatta, F., Ma, Z., van Giessen, J., Massalini, S., Lopez-Iglesias, C., van Son, G.J.F., DeMartino, J., Damen, J.M.A., et al. (2024). Human fetal brain self-organizes into long-term expanding organoids. Cell *187*, 712-732 e738. 10.1016/j.cell.2023.12.012.
2. Pellegrini, L., Bonfio, C., Chadwick, J., Begum, F., Skehel, M., and Lancaster, M.A. (2020). Human CNS barrier-forming organoids with cerebrospinal fluid production. Science *369*. 10.1126/science.aaz5626.
3. Zappaterra, M.D., Lisgo, S.N., Lindsay, S., Gygi, S.P., Walsh, C.A., and Ballif, B.A. (2007). A comparative proteomic analysis of human and rat embryonic cerebrospinal fluid. J Proteome Res *6*, 3537-3548. 10.1021/pr070247w.
4. Guo, L., Ren, H., Zeng, H., Gong, Y., and Ma, X. (2019). Proteomic analysis of cerebrospinal fluid in pediatric acute lymphoblastic leukemia patients: a pilot study. Onco Targets Ther *12*, 3859-3868. 10.2147/OTT.S193616.
5. Dayon, L., Cominetti, O., Wojcik, J., Galindo, A.N., Oikonomidi, A., Henry, H., Migliavacca, E., Kussmann, M., Bowman, G.L., and Popp, J. (2019). Proteomes of Paired Human Cerebrospinal Fluid and Plasma: Relation to Blood-Brain Barrier Permeability in Older Adults. J Proteome Res *18*, 1162-1174. 10.1021/acs.jproteome.8b00809.
6. Sarieva, K., Kagermeier, T., Khakipoor, S., Atay, E., Yentur, Z., Becker, K., and Mayer, S. (2023). Human brain organoid model of maternal immune activation identifies radial glia cells as selectively vulnerable. Mol Psychiatry *28*, 5077-5089. 10.1038/s41380-023-01997-1.
